# Mitochondrial Dynamics Tracking with Two-Photon Phosphorescent Terpyridyl Iridium(III) Complexes

**DOI:** 10.1038/srep20887

**Published:** 2016-02-11

**Authors:** Huaiyi Huang, Pingyu Zhang, Kangqiang Qiu, Juanjuan Huang, Yu Chen, Liangnian Ji, Hui Chao

**Affiliations:** 1 MOE Key Laboratory of Bioinorganic and Synthetic Chemistry, School of Chemistry and Chemical Engineering, Sun Yat-Sen University, Guangzhou 510275, P. R. China

## Abstract

Mitochondrial dynamics, including fission and fusion, control the morphology and function of mitochondria, and disruption of mitochondrial dynamics leads to Parkinson’s disease, Alzheimer’s disease, metabolic diseases, and cancers. Currently, many types of commercial mitochondria probes are available, but high excitation energy and low photo-stability render them unsuitable for tracking mitochondrial dynamics in living cells. Therefore, mitochondrial targeting agents that exhibit superior anti-photo-bleaching ability, deep tissue penetration and intrinsically high three-dimensional resolutions are urgently needed. Two-photon-excited compounds that use low-energy near-infrared excitation lasers have emerged as non-invasive tools for cell imaging. In this work, terpyridyl cyclometalated Ir(III) complexes (**Ir1-Ir3**) are demonstrated as one- and two-photon phosphorescent probes for real-time imaging and tracking of mitochondrial morphology changes in living cells.

Mitochondria are a type of organelle found in large numbers that function as the central hub of catabolic and anabolic metabolism within eukaryotic cells. Many important cellular events such as energy production, apoptosis regulation, redox balance, lipid modification, enzyme activity regulation, calcium balance, cell cycle, cell growth, cell differentiation, and innate immunity are all involved in the function of mitochondria^1^,^2^. Thus, mitochondria are expected to be vital platforms and drug targets for design of novel chemotherapeutic agents^3^,^4^. Mitochondria are dynamic organelles that frequently change their number, size, shape, and distribution within the cytoplasm in response to metabolic or environmental stresses. Mitochondria can enter constant dynamic cycles of fission (individual state) and fusion (network state) that construct the endoplasmic reticulum-mitochondria contacts^5^,^6^,^7^. When compromised by various injuries, solitary mitochondria are subjected to degradation. Cellular clearance of injured mitochondria relies on a self-eating process known as mitophagy^8^,^9^,^10^. Mitochondria and autophagy are elaborately linked via homeostatic elements that act in response to such significant cell environment changes as energy, nutrient, and oxidative stress. Recent studies revealed that defects in autophagic degradation of mitochondria are associated with neurodegenerative diseases, which highlights the importance of observing the dynamic morphologic changes in mitochondria^11^,^12^. Thus, direct visual observation of mitophagy events promises new insights into cellular activities that depend on mitochondria.

Fluorescence microscopy is a highly sensitive technique for detection and imaging of organelles in living cells. The resolution of fluorescent imaging can reach several hundred nanometers and, most importantly, this technique can be used to visualize morphological details in living cells that cannot be resolved by other technologies^13^. Many types of commercial mitochondrial organic dyes are available, such as Rhodamine 123, MitoTracker^®^ Green FM and MitoTracker^®^ Red FM. The applications of these mitochondrial dyes are somewhat limited due to their poor photostability, small Stokes shifts and need for high-energy excitation lasers^14^, and these dyes can be easily washed out of cells once the mitochondria membrane potential is lost. In addition, due to the accompanying concentration quenching effect, diluted solutions should be used (50–200 nM) during the cell imaging process, which results in low photostability. At high concentrations, these dyes tend to aggregate (quenching fluorescence) and stain other organelles^15^. However, a probe for real-time dynamic tracking of mitophagy *in vivo* requires more stringent properties, such as good biocompatibility, high mitochondria-targeting efficiency, superior photostability, deep tissue penetration and intrinsically high three-dimensional resolution.

Recently, cyclometalated iridium(III) complexes have gained increasing attention in bioimaging and biosensing due to their rich photophysical properties, e.g., large Stokes shifts, good biocompatibility, and high photostability^16^,^17^,^18^,^19^,^20^. Moreover, certain Ir(III) complexes exhibit strong two-photon phosphorescence and have been applied in biosensing and bioimaging^21^,^22^. Two-photon imaging offers properties superior to those of one-photon imaging with deeper tissue penetration, reduced photobleaching, reduced autofluorescence interference, and prolonged observation time^23^,^24^,^25^. To the best of our knowledge, although certain Ir(III) complexes can stain mitochondria^26^,^27^, the use of terpyridyl Ir(III) complexes [Ir(N^N^N)(C^N)Cl]^+^ (N^N^N = tridentate polypyridyl ligands; C^N = cyclometalated ligand, **Ir1**-**Ir3**, Fig. 1) for mitochondrial imaging in living cells has been rarely reported. Different from the conventional Ir(III) complexes motifs, [Ir(N^N^N)(C^N)Cl]^+^ is a type of Ir(III) architecture that exhibits excellent photophysical and photochemical properties and lacks in-depth biological investigation^28^,^29^,^30^. In this work, we report the use of **Ir1**-**Ir3** as one- and two-photon agents to track mitophagy in living cells and study the substituent influences of 4′-p-tolyl-2,2′:6,2″-terpyridine (ttpy) on phosphorescent properties, cell uptake efficiency, and mitochondria imaging ability.

## Results and Discussion

**Figure 2:**
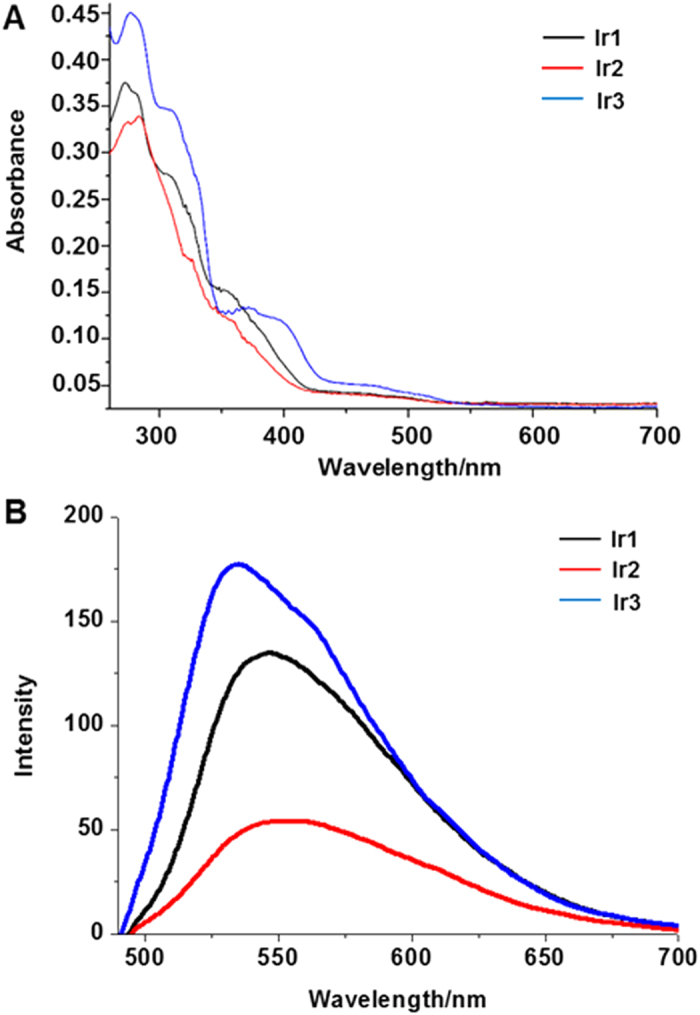
(**A**) UV-Vis absorption spectra of **Ir1-Ir3** (10 μM) in phosphate buffer solution (PBS, pH = 7.4) buffer. (**B**) Phosphorescence spectra of **Ir1-Ir3** (10 μM) in PBS buffer. The excitation wavelength for **Ir1-Ir3** is 458 nm.

The synthesis details and characterization of **Ir1**-**Ir3** are summarized in the Supporting Information (Figs S1–S6). The UV-Vis absorption spectra of **Ir1**-**Ir3** are presented in Fig. 2A and Fig. S7. These complexes exhibited intense high-energy absorption bands between 250 and 350 nm and weak absorption bands between 350 and 480 nm. Upon substitution of -H by –CH_3_ or –F, the UV-Vis spectrum exhibited a slight red shift accompanied by greater light absorbance. The greatest two-photon absorption (TPA) cross-section (δ) of **Ir1**-**Ir3** occurred at approximately 750 nm and was approximately 70−130 Göppert Mayer (GM) units (Fig. S8), a value that is much larger than those of the commercial MTR and MTG probes^31^. The phosphorescence spectra in H_2_O are shown in Fig. 2B, and **Ir1**-**Ir3** emitted yellow phosphorescence in water with quantum yields of approximately 0.10. In organic solvents CH_3_OH and CHCl_3_, the quantum yields were enhanced to approximately 0.20 (Fig. S9 and Table S1). All of the Ir(III) complexes exhibited large Stokes shifts (77–101 nm, λ_ex_ = 458 nm), which were similar to the other Ir(III) analogues^28^,^29^,^30^. Moreover, **Ir1**-**Ir3** showed excellent stability in water at room temperature in daylight, as determined by time-dependent UV-Vis spectra and fluorescent spectra (Fig. S10). The Cl ligand was coordinated so tightly to the Ir metal center that no apparent hydrolysis of Cl could be observed within 24 h, which matches previous reports^32^. In contrast, all of the commercial organic mitochondria dyes need to be carefully protected from light and stored at ≤ −20 °C 14. The TPA cross-section and excellent phosphorescent property rendered **Ir1**-**Ir3** suitable for bioimaging.

The lipophilicity of a compound is well known to have an important effect on cell uptake efficiency, and this lipophilicity can be quantitatively evaluated by log*P*_***o*****/*****w***_ values, where P is the partition coefficient between octanol and water. For high cell uptake compounds, the values range from −0.4 to 5.6, with an average of 2.52^33^,^34^. The log P_***o/w***_ values were detected by the “shake-flask” method. The complexes exhibited positive log*P*_***o*****/*****w***_ values for **Ir1** (1.3)**, Ir2** (0.9), and **Ir3** (1.5). It appeared that substitution on the ttpy ligand enhanced the lipophilicity, and a positive log *P*_*o*/*w*_ value might facilitate higher cell uptake ability and cell imaging efficiency.

**Figure 3:**
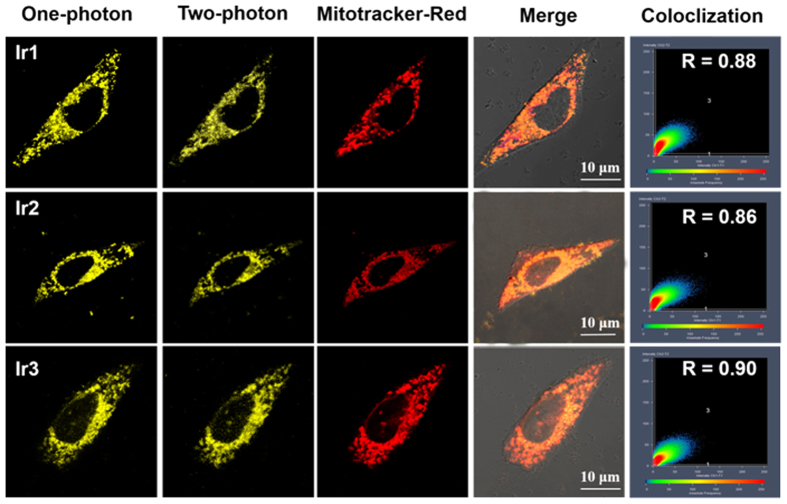
Co-localization images of **Ir1-Ir3** with MTR and co-localization coefficient (**R**). The one photon excitation wavelength for **Ir1-Ir3** is 458 nm and the two photon excitation wavelength for **Ir1-Ir3** is 750 nm. The excitation wavelength of MTR is 543 nm. Emission filter: 552 ± 20 nm (for **Ir1**), 547 ± 20 nm (for **Ir2**), 535 ± 20 nm (for **Ir3**), 640 ± 20 nm (for MTR). Scale bars = 10 μm.

Using laser scanning confocal microscopy, **Ir1**-**Ir3** were assessed for their ability to stain living cells. No nuclear uptake was observed. **Ir1**-**Ir3** was located mostly in the cytoplasm and specifically stained the thread-like organelles in HeLa cells (Fig. 3). The signal of **Ir1-Ir3** merged well with the commercial mitochondrial dye MTR. In contrast, using the other cytoplasm dyes, such as lysosome tracker LTR, no overlay signal could be observed (Fig. S11). The mitochondria-selective staining property of **Ir1-Ir3** was further confirmed using an inductively coupled plasma mass spectrometry (ICP-MS) assay (Fig. S12). The iridium concentration within mitochondria was significantly higher than that in the other organelles. The cell uptake efficiency of **Ir3** was better than **Ir1** and **Ir2,** as presented by the ICP-MS data. Moreover, at a concentration of 5 μM, the viability of HeLa cells treated with **Ir1**-**Ir3** was greater 80%, even after 6 h of incubation (Fig. S13).

Inspired by the excellent luminescence and mitochondria-targeting property of **Ir1-Ir3**, we further investigated the concentration-dependent mitochondria imaging efficiency of **Ir1-Ir3**. We found that at smaller concentrations from 0.1 to 2.5 μM, **Ir1-Ir3** retained the ability to target mitochondria (Fig. S14). These results revealed that **Ir1-Ir3** exhibit superior mitochondria imaging ability compared with that of MTR. The use of diluted solutions of MTR or MTG (50–200 nm) in the cell imaging process results in low photostability. However, at high concentrations, MTR or MTG tend to aggregate, which quenches the fluorescence and stains other organelles.

The cellular transport pathways of **Ir1-Ir3** were also investigated. Small molecules are generally taken up by living cells via an energy-dependent pathway (endocytosis, active transport) or an energy-independent (facilitated diffusion, passive diffusion) pathway^35^,^36^. HeLa cells were pretreated with the endocytosis inhibitor filipin, but this treatment it did not inhibit **Ir1-Ir3** staining mitochondria, indicating that **Ir1-Ir3** did not enter cells via the endocytosis pathway. However, HeLa cells that were pretreated with the metabolic inhibitors 2-deoxy-D-glucose and oligomycin or incubated at low temperature (4 °C) showed significantly diminished uptake of Ir(III) complexes. The intracellular phosphorescence apparently receded, which illustrated that **Ir1-Ir3** enter the cells via an energy-dependent pathway (Fig. S15).

In order to discuss the mechanism that **Ir1-Ir3** can selectively target mitochondria, we searched for more information on the structural character of mitochondria. The mitochondria are enclosed by two membranes. The mitochondrial external membrane separates the cell cytoplasm from its interior and possesses high membrane permeability. Mitochondria play important roles in maintaining genomic integrity because they continuously oxidize substrates and maintain a proton gradient across the lipid bilayer with a large mitochondrial membrane potential of close to −180 mV^37^,^38^. Therefore, cationic probes prefer to locate at the mitochondria than the other cellular component^39^,^40^,^41^. Some further reports mentioned that cationic species with a log P value between 0 and 5 tend to accumulate in the mitochondria^42^. The mechanism of mitochondria targeting ability may due to the lipophilicity and positive charge of **Ir1-Ir3**.

**Figure 4:**
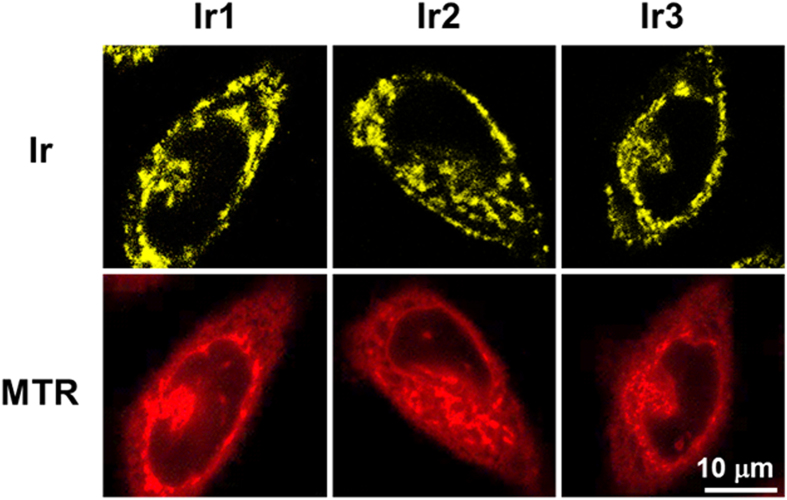
Images of CCCP pre-treated HeLa cells stained by Ir1-Ir3 and MTR. The excitation wavelength of MTR is 543 nm. Emission filter: 552 ± 20 nm (for **Ir1**), 547 ± 20 nm (for **Ir2**), 535 ± 20 nm (for **Ir3**), 640 ± 20 nm (for MTR). Scale bars = 10 μm.

Due to the membrane potential gradient, cationic mitochondria-specific dyes tend to accumulate in the mitochondria. However, under conditions of metabolic stress, drug treatment, radiation or autophagy, MTR tends to stain other cellular components. To access the tolerance of **Ir1-Ir3** to mitochondrial membrane potential change, carbonyl cyanide m-chlorophenylhydrazone (CCCP), an oxidative phosphorylation uncoupler that can abolish the mitochondrial membrane potential, was used to treat the cells prior to the staining procedure^43^. As shown in Fig. 4, the thread-like organelles tubular-like networks confirmed that **Ir1-Ir3** were capable of retaining mitochondria in CCCP-treated cells. In contrast, the commercially available mitochondrial imaging agents (e.g., MTR) tended to distribute at the cytoplasm and stain other cytoplasmic component in addition to mitochondria. The assembly of MTR in mitochondria is mainly dependent on the interaction with the mitochondrial internal membrane. Thus **Ir1-Ir3** may target mitochondria due to the mitochondrial potential but locate at different parts inside mitochondria compared with MTR.

**Figure 5:**
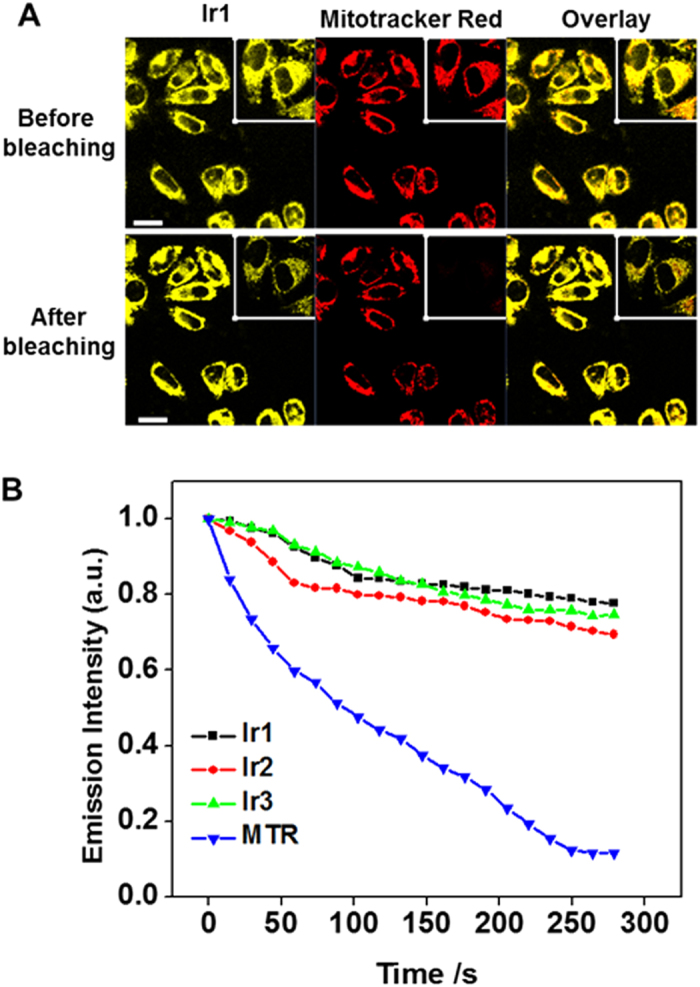
(**A**) Photobleaching assay of **Ir3** and MTR in HeLa cells. (**B**) Normalized emission intensity loss of **Ir1-Ir3** and MTR in HeLa cells with increasing bleaching time. Scale bars = 50 μm.

Photobleaching experiments were also performed to investigate the photostability of **Ir1**-**Ir3**. The fluorescence intensity of MTG and MTR decreased rapidly, whereas the signal loss of **Ir1**-**Ir3** was relatively small (Fig. 5). MTG or MTR completely lost their fluorescence signal after photobleaching, but all the Ir(III) complexes maintained greater than 50% intensity. The photobleaching assay revealed that **Ir1**-**Ir3** exhibit superior photostability compared with the MitoTracker dyes. Moreover, an efficient probe for image-tracking applications should minimally perturb living cell systems.

**Figure 6:**
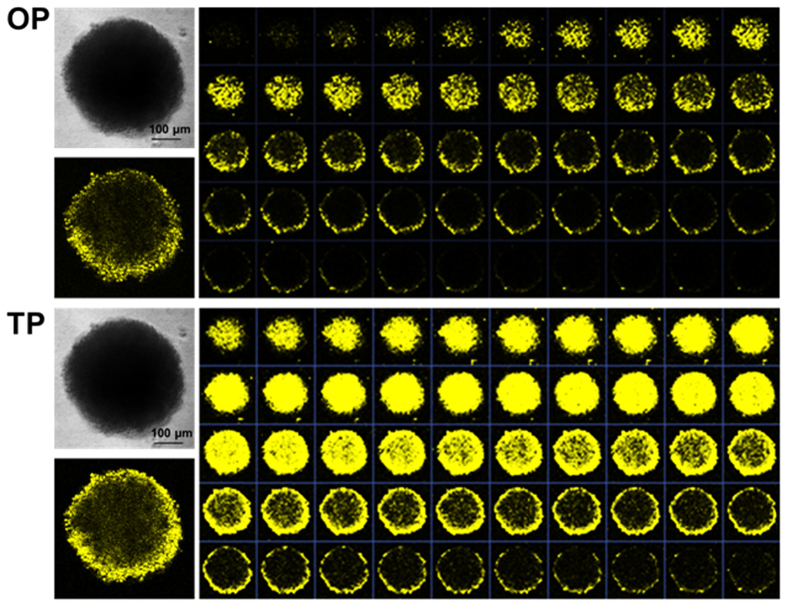
Axial OPM and TPM phosphorescence of Ir3 (5 μm) on intact 3D multicellular tumor spheroids. Scale bars = 100 μm.

Two-photon imaging uses low-energy near-infrared light excitation (700–1100 nm) and has emerged as a non-invasive tool for bioimaging. This technique presents several advantages over one-photon imaging, such as a deeper penetration depth, reduced photobleaching, reduced autofluorescence interference, and a prolonged observation time. The tubular-like mitochondrial branch network was clearly illuminated by **Ir1-Ir3** (Fig. 2 and Fig. S16) under two-photon excitation (750 nm). In contrast, MTR exhibited much smaller signals due to the small TPA cross-section. Traditional two-dimensional cultures of adherent cell monolayers are routinely used in biomedical and life science research. However, these cultures present significant limitations for reproducing the complexity and pathophysiology of tissue *in vivo*. Three-dimensional multi-cellular tumor spheroids (3D MCTSs) are heterogeneous cell aggregates that have been gradually accepted as a valid intermediate that bridges the gap between monolayer *in vitro* cells and *in vivo* tissue^44^,^45^,^46^,^47^. Because two-photon fluorescence microscopy (TPM) exhibits deeper tissue penetration depths compared with one-photon fluorescence microscopy (OPM), 3D MCTSs were used to confirm the penetration depth of **Ir1-Ir3**. As shown in Fig. 6 and Figs S17 and 18, **Ir1-Ir3** were capable of penetrating deep into the HeLa MCTSs, up to ~120 μm from the periphery, and the internal structure of the MCTSs was illuminated using two-photon excitation. In contrast, a weak luminescence intensity was observed on the surface of the spheroids up to a depth of ~50 μm for OPM imaging. Thus, the spheroids exhibited much stronger phosphorescence in the deeper sections of the MCTSs with two-photon laser excitation compared with one-photon excitation. The signal from one-photon probes, such as commercial MitoTracker dyes, exhibits poor tissue penetration, thus restricting their application to cell monolayers (Fig. S19). The phosphorescence images were captured every 4 μm along the Z-axis. Therefore, **Ir1-Ir3** were demonstrated as excellent two-photon probes for mitochondrial staining with deep tissue penetration.

**Figure 7:**
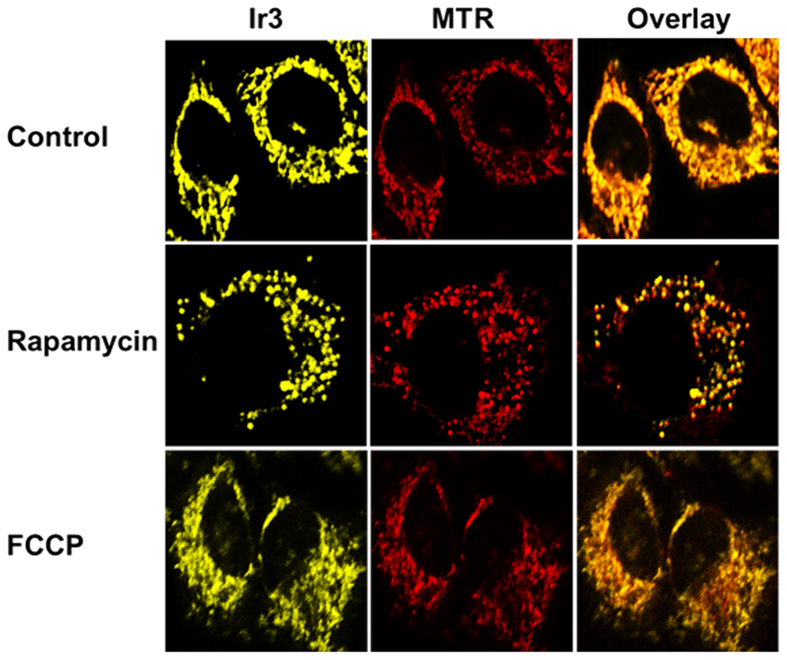
Cell imaging of Ir3 and MTR within living HeLa cells treated with rapamycin (500 nm) and FCCP. Scale bars = 10 μm.

The word mitochondrion comes from the Greek meaning “thread” and “grain-like”. The constant dynamic cycles of mitochondrial fission (individual state) and mitochondrial fusion (network state) construct the endoplasmic reticulum-mitochondria contacts. Moreover, mitochondria continuously fuse and fragment during autophagy and uncoupling. Rapamycin, an mTOR inhibitor, has been shown to extend the lifespan and increase the level of autophagy, which is implicated upstream of the mitochondrial fragmentation^48^,^49^. In this work, **Ir3** was selected to visualize rapamycin (500 nm, 12 h) and FCCP (10 *μ*M, 12 h) induced mitochondrial fission and fusion. As shown in Fig. 7, the mitochondrial morphology of fission and fusion induced by rapamycin was clearly illuminated by **Ir3** in HeLa cells using two-photon excitation. Additionally, mitochondrial uncouplers, such as FCCP (carbonilcyanide p-triflouromethoxy-phenylhydrazone), did not reduce the viability of HeLa cells but could induce large-scale fission of mitochondria^50^. FCCP is referred to as an uncoupling agent because it disrupts ATP synthesis and inhibits mitochondrial potential. In mammalian cells, loss of mitochondrial membrane potential causes mitophagy, and the most rapid fission of mitochondria was induced by uncouplers (FCCP). In Fig. 7, we show that FCCP did not reduce the viability of HeLa after 12 h of treatment, and the fragmented mitochondria gathered near the nucleus.

Mitochondria and autophagy are elaborately linked homeostatic elements that act in response to significant cell environment changes, such as energy, nutrient, and oxidative stresses. Recent studies reveal that defects in autophagic degradation of mitochondria are associated with neurodegenerative diseases, thus highlighting the importance of observing the dynamic morphologic changes of mitochondria. When compromised by various injuries, solitary mitochondria are subject to degradation. The term mitophagy has been introduced for the specific process of mitochondrial autophagy. Mitochondria are a major target of autophagy because they occupy nearly 20% of the cytoplasmic volume even in well-nourished young animals. Mitophagy aids in clearing mitochondria with mutations of mitochondrial DNA and damaged mitochondria^51^,^52^,^53^,^54^,^55^.

**Figure 8:**
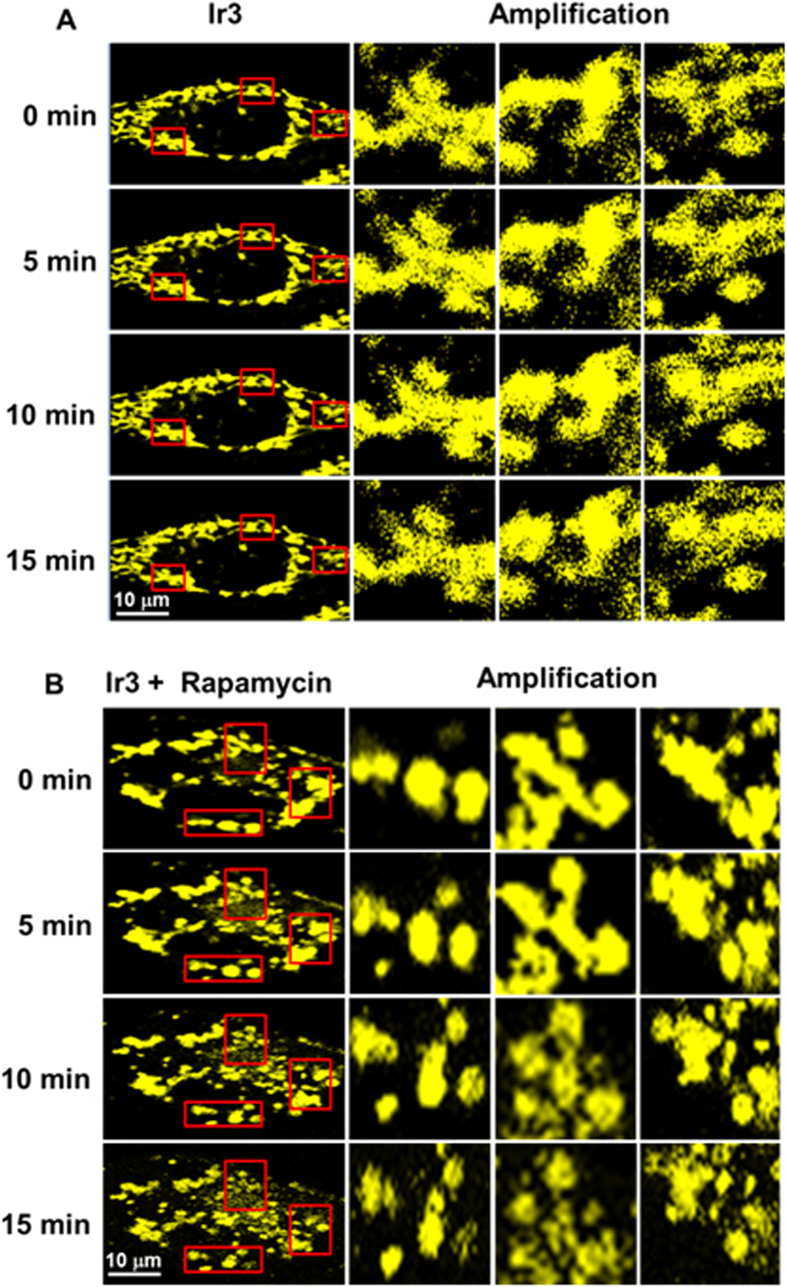
(**A**) Real-time tracking of mitochondrial dynamic in living HeLa stained with **Ir3** (5 μM, 1 h). (**B**) Real-time monitoring of mitochondrial morphology changes during incubation with rapamycin. HeLa cells were pre-stained with **Ir3.** Scale bars = 10 μm.

We used **Ir3** to monitor mitochondrial dynamics and mitophagy in real time under two-photon excitation. HeLa cells were pre-stained with **Ir3**, and the cells were incubated in DMEM medium with different concentrations of glucose (4500 mg/L glucose for mitochondrial dynamic tracking and 1000 mg/L glucose with 500 nm rapamycin to induce mitophagy). Normal mitochondrial dynamics, including fusion and fission, continuously occurred and could be easily recognized after **Ir3** staining (Fig. 8A). In the presence of low glucose concentration, the rod-like mitochondria tended to degrade and became small dots. It has been reported that hunger treatment (low glucose) or rapamycin will induce mitochondrial autophagy to maintain the cellular energy supply^56^. Thus, **Ir3** enabled direct visualization of the quick action of mitochondrial elongation or contraction and mitophagy.

## Conclusion

In summary, we have designed and developed a new series of iridium(III) complexes, known as **Ir1-Ir3**, that exhibit one- and two-photon phosphorescence, high quantum yields, large two-photon absorption cross-sections, and mitochondria-specific staining properties. These complexes can clearly image the inner structure of 3D multicellular tumor spheroids with deeper tissue penetration under two-photon excitation. Among the whole complexes, the –F substituted complex **Ir3** exhibited properties superior to those of the other two complexes. We successfully used **Ir3** to track mitochondrial dynamics and mitophagy in real time in living cells. **Ir3** can greatly simplify the staining procedures that are used to study the key events associated with mitochondrial fission and fusion and can aid in developing an understanding of the role of mitochondrial fission and fusion and mitophagy in physiological cell function and dysfunction. With an improved understanding of mitochondrial morphology changes, new techniques for real-time tracking of mitochondria in living cells can enable a range of new diagnoses and therapies.

## Methods

All solvents were of analytical grade. All buffer components were of biological grade and used as received. Iridium chloride hydrate, p-tolualdehyde and 2-acetylpyridine, 2-phenyl-pyridine (ppy), cisplatin, rapamycin, carbonyl cyanide-4-(trifluoromethoxy)-phenylhydra-zone (FCCP) and carbonyl cyanide m-chlorophenyl-hydrazone (CCCP) were bought from Sigma Aldrich (USA). MitoTracker Red FM (MTR) was purchased from Life Technologies (USA). Microanalysis (C, H, and N) was performed using a Vario EL elemental analyzer. 1H NMR spectra were recorded on a Bruker ADVANCE AV 400 NMR spectrometer using (CD_3_)_2_SO as a solvent at room temperature and TMS as the internal standard. Electrospray mass spectra (ES-MS) were recorded on a LCQ system (Finnigan MAT, USA). The spray voltage, tube lens offset, capillary voltage and capillary temperature were set at 4.50  kV, 30.00 V, 23.00 V and 200 °C, respectively, and the quoted m/z values refer to the major peaks in the isotope distribution. UV–Vis spectra were recorded on a Perkin-Elmer Lambda 850 spectrophotometer. Emission spectra were recorded on a Perkin-Elmer LS 55 spectrofluorophotometer at room temperature.

### Synthesis of Ir(III) complexes.

4′-p-tolyl-2,2′:6,2″-terpyridine (ttpy), 4′-p-methyltolyl-2,2′:6,2″-terpyridine (mettpy), 4′-p-fluorotolyl-2,2′:6,2″-terpyridine (fttpy) were prepared as described by Wang and Hanan^13^ Ir(ttpy)Cl_3_, Ir(mettpy)Cl_3_ and Ir(fttpy)Cl_3_ were prepared based on the procedure reported by Chirdon *et al*.^13^.

### Synthesis of [Ir(mettpy)(ppy)Cl](PF_6_) (Ir1)

A mixture of [Ir(ttpy)Cl_3_] (124 mg, 0.20 mmol) and excess ppy (62 mg, 0.40 mmol) in glycol (10 mL) was stirred overnight at 180 °C under Ar and became a clear solution. After cooling to room temperature, the solution was poured into 50 mL saturated NH_4_PF_6_ solution to obtain a precipitate. The precipitate was isolated by filtration and washed with water and diethyl ether 3 times. The crude products were purified by column chromatography on alumina using acetonitrile−toluene as the eluent. The solvent was removed under reduced pressure, and purified yellow to orange Ir(III) complexes were obtained. Yield: 62%. Anal. calcd. for C_33_H_25_ClF_6_IrN_4_P: C, 46.56; H, 3.08; N, 6.58. Found: C, 46.47; H, 3.15; N, 6.43. ES-MS(CH_3_OH): m/z 705.0 [M-PF_6_^−^]^+^. ^1^H NMR (300 MHz, d_6_-DMSO) δ 9.87 (d, *J* = 5.7 Hz, 1H), 9.24 (s, 2H), 8.96 (d, *J* = 6.9 Hz, 2H), 8.49 (d, *J* = 7.2 Hz, 1H), 8.34 − 8.17 (m, 5H), 7.93 (d, *J* = 7.8 Hz, 1H), 7.80 (t, *J* = 6.6 Hz, 1H), 7.68 (d, *J* = 5.1 Hz, 2H), 7.54 (m, 4H), 6.91 (t, *J* = 7.5 Hz, 1H), 6.74 (t, *J* = 7.5 Hz, 1H), 6.07 (d, *J* = 7.6 Hz, 1H), 2.29 (s, 3H).

### Synthesis of [Ir(ttpy)(ppy)Cl](PF_6_) (Ir2)

A mixture of [Ir(ttpy)Cl_3_] (121 mg, 0.20 mmol) and excess ppy (62 mg, 0.40 mmol) was prepared in glycol (10 mL) solution. After cooling to room temperature, the solution was poured into 50 mL saturated NH_4_PF_6_ solution to obtain precipitates. The precipitate was isolate by filtration and washed with water and diethyl ether 3 times. The crude products were purified by column chromatography on alumina using acetonitrile−toluene as the eluent. The solvent was removed under reduced pressure, and purified yellow to orange Ir(III) complexes were obtained. Yield: 61%. Anal. calcd. for C_32_H_23_ClF_6_IrN_4_P: C, 45.91; H, 2.89; N, 6.69. Found: C, 45.79; H, 2.97; N, 6.55. ES-MS (CH_3_OH): m/z 691.0 [M-PF_6_^−^]^+^. ^1^H NMR (300 MHz, d_6_-DMSO) δ = 9.88 (d, *J* = 5.1 Hz, 1H), 9.26 (s, 2H), 8.96 (d, *J* = 8.1 Hz, 2H), 8.49 (d, *J* = 8.1 Hz, 1H), 8.33 − 8.12 (m, 3H), 8.26 − 8.19 (d, *J* = 7.2 Hz, 2H), 7.94 (d, *J* = 7.5 Hz, 1H), 7.85 − 7.78 (t, *J* = 6.9 Hz, 1H), 7.69 (m, 5H), 7.59 − 7.49 (t, *J* = 7.2 Hz, 2H), 6.92 (t, *J* = 7.5 Hz, 1H), 6.75 (t, *J* = 7.2 Hz, 1H), 6.07 (d, *J* = 8.1 Hz, 1H).

### Synthesis of [Ir(fttpy)(ppy)Cl](PF_6_) (Ir3)

A mixture of [Ir(fttpy)Cl_3_] (125 mg, 0.20 mmol) and excess ppy (62 mg, 0.40 mmol) in glycol (10 mL) was stirred overnight at 180 °C under Ar and became a clear solution. After cooling to room temperature, the solution was poured into 50 mL saturated NH_4_PF_6_ solution to obtain a precipitate. The precipitate was isolated by filtration and washed with water and diethyl ether 3 times. The crude products were purified by column chromatography on alumina using acetonitrile−toluene as the eluent. The solvent was removed under reduced pressure, and purified yellow to orange Ir(III) complexes were obtained. Yield: 65%. Anal. calcd. for C_32_H_22_ClF_7_IrN_4_P: C, 44.94; H, 2.71; N, 6.55. Found: C, 45.09; H, 2.67; N, 6.41. ES-MS(CH_3_OH): m/z 709.0 [M-PF_6_^−^]^+^. ^1^H NMR (300 MHz, d_6_-DMSO) δ 9.87 (d, *J* = 5.4 Hz, 1H), 9.25 (s, 2H), 8.94 (d, *J* = 7.8 Hz, 2H), 8.49 (d, *J* = 8.1 Hz, 1H), 8.45 − 8.33 (m, 2H), 8.33 − 8.25 (d, *J* = 7.2 Hz, 1H), 8.22 (t, *J* = 7.2 Hz, 2H), 7.94 (d, *J* = 7.9 Hz, 1H), 7.85 − 7.76 (t, *J* = 6.9 Hz, 1H), 7.69 (d, *J* = 5.4 Hz, 2H), 7.62 (t, *J* = 8.7 Hz, 2H), 7.57 − 7.50 (t, *J* = 6.3 Hz, 2H), 6.92 (t, *J* = 7.2 Hz, 1H), 6.74 (t, *J* = 7.2 Hz, 1H), 6.07 (d, *J* = 7.8 Hz, 1H).

### Determination of two-photon absorption cross-sections

The two-photon absorption (TPA) spectra of the complexes were determined over a broad spectral region using a two-photon induced luminescence (TPL) method relative to Rhodamine B in methanol as the standard. The two-photon luminescence data were acquired using an Opolette^TM^ 355II instrument (pulse width ≤ 100 fs, 80 MHz repetition rate, tuning range 710–840 nm, Spectra Physics, Inc., USA). Two-photon luminescence measurements were performed in fluorometric quartz cuvettes. The experimental luminescence excitation and detection conditions were conducted with negligible reabsorption processes, which can affect TPA measurements. The quadratic dependence of the two-photon induced luminescence intensity on the excitation power was verified at an excitation wavelength of 750 nm. The two-photon absorption cross-section of the complex was calculated at each wavelength according to Equation (1)^57^:


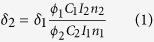


where *I* is the integrated luminescence intensity, *C* is the concentration, *n* is the refractive index, and *φ* is the quantum yield. The subscript ‘1’ refers to the reference samples, and ‘2’ indicates the experimental samples.

### Cell culture

Human cervix adenocarcinoma cells (HeLa) were obtained from the Experimental Animal Center of Sun Yat-sen University (Guangzhou, China). HeLa were cultured in Dulbecco’s modified Eagle medium supplemented with 10% FBS, penicillin (100 units/mL), and streptomycin (100 μg/mL) in a 5% CO_2_ humidified incubator at 37 °C.

### Cell viability assay

The cytotoxicity of the tested compounds toward HeLa cells was determined via MTT assay. Cells cultured in 96-well plates were grown to confluence. The compounds were dissolved in DMSO (0.1%, v/v) and immediately diluted with fresh media. The cells were incubated with **Ir1-Ir3** (5 μM) for 0.5 h, 1 h, 3 h and 6 h. The medium was removed, the cells were rinsed once with PBS, and fresh medium was applied. An amount of 15 μL of MTT solution (5 mg/mL) was added to each well, and the plates were incubated for an additional 4 h. The medium was carefully removed and DMSO was added (150 μL per well). The plate was incubated for 10 min with shaking. The absorbance at 595 nm was measured using a microplate reader (Infinite M200 Pro, Tecan, Männedorf, Switzerland).

### Cellular localization assay

Cells were incubated with **Ir1-Ir3** (5 μM) at 37 °C for 0.5 h and further co-incubated with MTR (150 nm) at 37 °C for another 0.5 h. Cells were washed three times with ice-cold PBS and visualized immediately using confocal microscopy (LSM 710, Carl Zeiss, Göttingen, Germany) with a 63× oil-immersion objective lens. The excitation wavelengths for one- and two-photon excitation of **Ir1-Ir3** are 458 nm and 750 nm, respectively. The excitation wavelength of MTR is 543 nm. Emission filter: 552 ± 20 nm (for **Ir1**), 547 ± 20 nm (for **Ir2**), 535 ± 20 nm (for **Ir3**), 640 ± 20 nm (for MTR).

### Cellular uptake assay

Cells were detached from the culture and preincubated with endocytosis inhibitors (200 μg/mL of filipin) and metabolic inhibitors (50 mM of 2-deoxy-D-glucose and 5 μM of oligomycin) for 1 h before incubation with 5 μM of **Ir1-Ir3** with inhibitor in fresh media for 1 h at 4 °C or 37 °C. After exposure to the Ir(III) complexes and the inhibitors for the desired time, the cells were washed with a PBS solution and were subjected to confocal luminescence imaging.

### ICP-MS assay

Exponentially growing HeLa cells were harvested, and the resulting single-cell suspension was plated into 100 mm tissue culture plates (Costar). After 24 h at 37 °C, the cells were incubated with 5 μM **Ir1-Ir3** complexes for 1 h at 37 °C in either medium with serum or medium without serum. The cells were rinsed with PBS, detached with trypsin, counted and divided into three portions. In the first portion, the nuclei were extracted using a nucleus extraction kit (Pierce, Thermo) following the manufacturer’s protocol. In the second portion, the cytoplasm was extracted using a cytoplasm extraction kit (Pierce, Thermo). In the third portion, the mitochondria were extracted using a mitochondrial extraction kit (Pierce, Thermo). The samples were digested with 60% HNO_3_ at RT for one day. Each sample was diluted with MilliQ H_2_O to obtain 2% HNO_3_ sample solutions. The iridium content was measured using inductively coupled plasma mass spectrometry (ICP-MS Thermo Elemental Co., Ltd.). Data were reported as the means ± standard deviation (n = 3).

### Photobleaching assay in living cells

In order to test the photostability of **Ir1-Ir3** as mitochondrial probes in cells, the one-photon fluorescence images were collected every 7 s in the channel. The excitation wavelength of MTR is 543 nm. Emission filter: 552 ± 20 nm (for **Ir1**), 547 ± 20 nm (for **Ir2**), 535 ± 20 nm (for **Ir3**), 640 ± 20 nm (for MTR). The fluorescence intensity of the images was recorded as well and compared with the initial intensity.

### Generation and standing of 3D MCTSs

MCTSs were cultured using the liquid overlay method^58^. HeLa cells in the exponential growth phase were dissociated by trypsin/EDTA solution to obtain single-cell suspensions. A number of 2500 diluted HeLa cells were transferred to 1% agarose-coated transparent 96-well plates with 200 μL of Dulbecco’s modified Eagle medium (DMEM) containing 10% serum. The single cells generated MCTSs approximately 400 μm in diameter at day 4 at 5% CO_2_ in air at 37 °C. For staining experiments, 5 μM (**Ir1-Ir3**) or 150 nM (MTR) in DMSO solution were incubated with MCTSs for 6 h. Cells were immediately visualized by confocal microscopy (LSM 710, Carl Zeiss, Göttingen, Germany) with a 10× objective lens. The excitation wavelengths for one- and two-photon excitation of **Ir1-Ir3** are 458 nm and 750 nm, respectively. The excitation wavelength of MTR is 543 nm. Emission filter: 552 ± 20 nm (for **Ir1**), 547 ± 20 nm (for **Ir2**), 535 ± 20 nm (for **Ir3**), 640 ± 20 nm (for MTR).

### Mitochondrial dynamics real-time tracking

HeLa cells were pretreated with **Ir3** (5 μM, 1 h), followed by replacement of the medium with fresh culture medium or low glucose culture medium together with rapamycin (500 nM) and incubation in a humidified incubator. Cell imaging was performed by confocal microscopy, and photographs were taken at a different time. Emission was collected at 551 ± 20 nm upon excitation at 750 nm by two-photon modes (LSM 710, Carl Zeiss, Göttingen Germany).

## Additional Information

**How to cite this article**: Huang, H. *et al*. Mitochondrial Dynamics Tracking with Two-Photon Phosphorescent Terpyridyl Iridium(III) Complexes. *Sci. Rep*. **6**, 20887; doi: 10.1038/srep20887 (2016).

## Supplementary Material

Supplementary Information

## Figures and Tables

**Figure 1:**
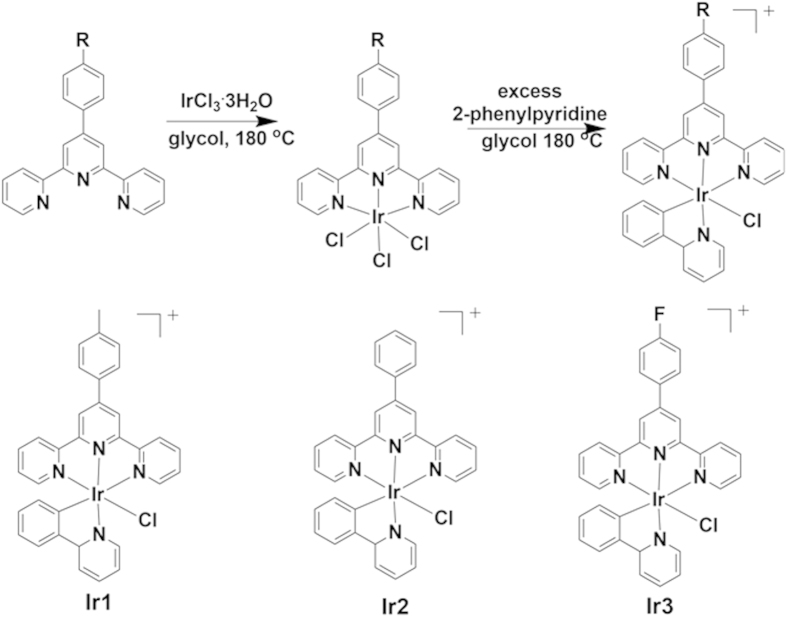
Chemical structures of Ir(III) complexes Ir1-Ir3.

## References

[b1] Henze K. & Martin W. Evolutionary biology: essence of mitochondria. Nature 426, 127–128 (2002). 14614484 10.1038/426127a

[b2] Green D. R. Apoptotic pathways: paper wraps stone blunts scissors. Cell 94, 695–698 (1998). 10929706 10.1016/s0092-8674(00)00003-9

[b3] Fulda S., Galluzzi L. & Kroemer G. Targeting mitochondria for cancer therapy. Nat. Rev. Drug Discov. 9, 447–464 (2010). 20467424 10.1038/nrd3137

[b4] Hoye A. T. . Targeting mitochondria. Acc. Chem. Res. 41, 87–97, (2008). 18193822 10.1021/ar700135m

[b5] Bossy-Wetzel E. . Mitochondrial fission in apoptosis, neurodegeneration and aging. Curr. Opin. Cell Biol. 15, 706–716 (2003). 14644195 10.1016/j.ceb.2003.10.015

[b6] Liesa M., Palacín M. & Zorzano A. Mitochondrial dynamics in mammalian health and disease. Physiol. Rev. 89, 799–845 (2009). 19584314 10.1152/physrev.00030.2008

[b7] Berman S. B., Pineda F. J. & Hardwick J. M. Mitochondrial fission and fusion dynamics: the long and short of it. Cell Death Differ. 15, 1147–1152 (2008). 18437161 10.1038/cdd.2008.57 PMC2614113

[b8] Geisler S. . PINK1/Parkin-mediated mitophagy is dependent on VDAC1 and p62/SQSTM1. Nat. Cell Biol. 12, 119–131, (2010). 20098416 10.1038/ncb2012

[b9] Zhang J. & Ney P. A. Role of BNIP3 and NIX in cell death, autophagy, and mitophagy. Cell Death Differ. 16, 939–946 (2009). 19229244 10.1038/cdd.2009.16 PMC2768230

[b10] Narendra D. . p62/SQSTM1 is required for Parkin-induced mitochondrial clustering but not mitophagy; VDAC1 is dispensable for both. Autophagy 6, 1090–1106 (2010). 20890124 10.4161/auto.6.8.13426 PMC3359490

[b11] Wong E. & Cuervo A. M. Autophagy gone awry in neurodegenerative diseases. Nat Neurosci. 13, 805–811 (2010). 20581817 10.1038/nn.2575 PMC4038747

[b12] Geisler S. . PINK1/Parkin-mediated mitophagy is dependent on VDAC1 and p62/SQSTM1. Nat Cell Biol. 12, 119–131 (2010). 20098416 10.1038/ncb2012

[b13] Yang Y. M. . Luminescent chemodosimeters for bioimaging. Chem. Rev. 2013, 113, 192–270 (2009). 22702347 10.1021/cr2004103

[b14] Johnson I. & Spence M. T. Z. The Molecular Probes Handbook, 11th ed.; Life Technologies Corporation: Carlsbad, CA, (2010).

[b15] Leung W. T. . A photostable AIE luminogen for specific mitochondrial imaging and tracking. J. Am. Chem. Soc. 135, 62–65 (2013). 23244346 10.1021/ja310324q

[b16] Zhao Q., Huang C. H. & Li F. Y. Phosphorescent heavy-metal complexes for bioimaging. Chem. Soc. Rev. 40, 2508–2524 (2011). 21253643 10.1039/c0cs00114g

[b17] Chen Z. Q. . Functional IrIII complexes and their applications. Adv. Mater. 22, 1534–1539 (2010). 20437503 10.1002/adma.200903233

[b18] You Y. & Nam W. Photofunctional triplet excited states of cyclometalated Ir (III) complexes: beyond electroluminescence. Chem. Soc. Rev. 41, 7061–7084 (2012). 22797418 10.1039/c2cs35171d

[b19] Ma D. L. . Bioactive Luminescent Transition-Metal Complexes for Biomedical Applications. Angew. Chem. Int. Ed. 52, 7666–7682 (2013). 10.1002/anie.201208414 23765907

[b20] Qiu K. Q. . Mitochondria-specific imaging and tracking in living cells with two-photon phosphorescent iridium (iii) complexes. J. Mater. Chem. B 3, 6690–6697 (2015). 10.1039/c5tb01091h 32262803

[b21] Xu W. J. . Rational Design of Phosphorescent Chemodosimeter for Reaction-Based One-and Two-Photon and Time-Resolved Luminescent Imaging of Biothiols in Living Cells. Adv. Healthc. Mater. 3, 658–669 (2014). 24243822 10.1002/adhm.201300278

[b22] Boreham E. M. . A cyclometallated fluorenyl Ir(III) complex as a potential sensitiser for two-photon excited photodynamic therapy (2PE-PDT). Dalton Trans. 44, 16127–16135 (2015). 26289593 10.1039/c5dt01855b

[b23] Bae S. K. . A ratiometric two-photon fluorescent probe reveals reduction in mitochondrial H2S production in Parkinson’s disease gene knockout astrocytes. J. Am. Chem. Soc. 135, 9915–9923 (2013). 23745510 10.1021/ja404004v

[b24] Huang H. Y. . Highly Charged Ruthenium (II) Polypyridyl Complexes as Lysosome-Localized Photosensitizers for Two-Photon Photodynamic Therapy. Angew. Chem. Int. Ed. 127, 14255–14258 (2015). 10.1002/anie.201507800 26447888

[b25] Zhang P. . RuNH 2@ AuNPs as two-photon luminescent probes for thiols in living cells and tissues. Biomaterials 35, 9003–9011 (2014). 25103232 10.1016/j.biomaterials.2014.07.021

[b26] Chen Y. . Phosphorescent iridium (III) complexes as multicolor probes for specific mitochondrial imaging and tracking. Biomaterials 35, 2–13 (2014). 24120043 10.1016/j.biomaterials.2013.09.051

[b27] Chen Y. . Mitochondria-specific phosphorescent imaging and tracking in living cells with an AIPE-active iridium (III) complex. Chem. Commun. 49, 11095–11097 (2013). 10.1039/c3cc46957c 24141977

[b28] Wang J. & Hanan G. S. A facile route to sterically hindered and non-hindered 4′-aryl-2, 2′: 6′, 2-terpyridines. Synlett. 8, 1251–1254 (2005).

[b29] Chirdon D. N. . [Ir (N^N^N)(C^N) L]^+^: A New Family of Luminophores Combining Tunability and Enhanced Photostability. Inorg Chem. 53, 1487–1499 (2014). 24437359 10.1021/ic402411g

[b30] Sato S. . A highly efficient mononuclear iridium complex photocatalyst for CO2 reduction under visible light. Angew. Chem. Int. Ed. 52, 988–992 (2013). 10.1002/anie.201206137 23197479

[b31] Miao F. . Novel fluorescent probes for highly selective two-photon imaging of mitochondria in living cells. Biosens Bioelectron. 55, 423–429 (2014). 24441022 10.1016/j.bios.2013.12.044

[b32] Obara S. . Highly Phosphorescent Iridium Complexes Containing Both Tridentate Bis(benzimidazolyl)-benzene or -pyridine and Bidentate Phenylpyridine: Synthesis, Photophysical Properties, and Theoretical Study of Ir-Bis(benzimidazolyl)benzene Complex. Inorg. Chem. 45, 8907–8921 (2006). 17054350 10.1021/ic060796o

[b33] Zheng Y. R. . Pt (IV) prodrugs designed to bind non-covalently to human serum albumin for drug delivery. J. Am. Chem. Soc. 136, 8790–8798 (2014). 24902769 10.1021/ja5038269 PMC4076294

[b34] Huang H. Y. . Targeting nucleus DNA with a cyclometalated dipyridophenazineruthenium (II) complex. J. Med. Chem. 57, 8971–8983 (2014). 25313823 10.1021/jm501095r

[b35] Li C. . A nonemissive iridium (III) complex that specifically lights-up the nuclei of living cells. J. Am. Chem. Soc. 133, 11231−11239 (2011). 21682270 10.1021/ja202344c

[b36] Puckett C. A. & Barton J. K. Mechanism of Cellular Uptake of a Ruthenium Polypyridyl Complex. Biochemistry 47, 11711−11716 (2008). 18855428 10.1021/bi800856t PMC2747514

[b37] Ly D. R. . The mitochondrial membrane potential (Δψm) in apoptosis; an update. Apoptosis 8, 115–128 (2003). 12766472 10.1023/a:1022945107762

[b38] Chen L. B. Mitochondrial membrane potential in living cells. Ann. Rev. Cell BioI. 4, 155–181 (1988). 10.1146/annurev.cb.04.110188.001103 3058159

[b39] Miao F. . Novel fluorescent probes for highly selective two-photon imaging of mitochondria in living cells. Biosens. Bioelectron. 55, 423–429 (2014). 24441022 10.1016/j.bios.2013.12.044

[b40] Sun Y. Q. . A Mitochondria-Targetable Fluorescent Probe for Dual-Channel NO Imaging Assisted by Intracellular Cysteine and Glutathione. J. Am. Chem. Soc. 136, 12520–12523 (2014). 25122520 10.1021/ja504156a

[b41] Hu Q. . Mitochondria-Targeted Cancer Therapy Using a Light-Up Probe with Aggregation-Induced-Emission Characteristics. Angew. Chem. Int. Ed. 53, 14225–14229 (2014) 10.1002/anie.201408897 25318447

[b42] Rashid F., Horobin R. W. & Williams M. A. Predicting the behaviour and selectivity of fluorescent probes for lysosomes and related structures by means of structure-activity models. Histochem. J. 23, 450–459 (1991). 1743993 10.1007/BF01041375

[b43] Heytler P. G. Uncoupling of oxidative phosphorylation by carbonyl cyanide phenylhydrazones. I. Some characteristics of m-CI-CCP action on mitochondria and chloroplasts. Biochemistry 2, 357–361 (1963). 13954345 10.1021/bi00902a031

[b44] Pampaloni F. . The third dimension bridges the gap between cell culture and live tissue. Nat. Rev. Mol. Cell Biol. 8, 839–845 (2007). 17684528 10.1038/nrm2236

[b45] Huang H. Y. . Comparison Between Polypyridyl and Cyclometalated Ruthenium (II) Complexes: Anticancer Activities Against 2D and 3D Cancer Models. Chem. Eur. J. 21, 715–725 (2015). 25388328 10.1002/chem.201404922

[b46] Mazzoleni G., Lorenzo D. & Steimberg N. Modelling tissues in 3D: the next future of pharmaco-toxicology and food research? Genes Nutr. 4, 13–22 (2009). 19104883 10.1007/s12263-008-0107-0 PMC2654048

[b47] Hutchinson L. & Kirk R. High drug attrition rates-where are we going wrong? Nat. Rev. Clin. Oncol. 8, 189–190 (2011). 21448176 10.1038/nrclinonc.2011.34

[b48] Gomes L. C. . Mitochondrial morphology in mitophagy and macroautophagy. BBA-Mol. Cell. Res., 1833, 205–212 (2013). 10.1016/j.bbamcr.2012.02.012 22406072

[b49] Singh S. B. . Human IRGM regulates autophagy and cell-autonomous immunity functions through mitochondria. Nat. Cell Biol. 12, 1154–1165 (2010). 21102437 10.1038/ncb2119 PMC2996476

[b50] Pletjushkina O. Y. Effect of oxidative stress on dynamics of mitochondrial reticulum. BBA-Bioenergetics 1757, 518–524 (2006). 16829229 10.1016/j.bbabio.2006.03.018

[b51] Ashrafi G. & Schwarz T. L. The pathways of mitophagy for quality control and clearance of mitochondria. Cell Death Differ. 20, 31–42 (2013). 22743996 10.1038/cdd.2012.81 PMC3524633

[b52] Menzies R. A. & Gold P. H. The turnover of mitochondria in a variety of tissues of young adult and aged rats. J. Biol. Chem. 246, 2425–2429 (1971). 5553400

[b53] Brunk U. T. & Terman A. The mitochondrial-lysosomal axis theory of aging. Eur. J. Biochem. 269, 1996–2002 (2002). 11985575 10.1046/j.1432-1033.2002.02869.x

[b54] Lemasters J. J. Selective mitochondrial autophagy, or mitophagy, as a targeted defense against oxidative stress, mitochondrial dysfunction, and aging. Rejuvenation Res. 8, 3–5 (2005). 15798367 10.1089/rej.2005.8.3

[b55] Elmore S. P. . The mitochondrial permeability transition initiates autophagy in rat hepatocytes. FASEB J. 15, 2286–2287 (2001). 11511528 10.1096/fj.01-0206fje

[b56] Rodriguez-Enriquez S. . Tracker dyes to probe mitochondrial autophagy (mitophagy) in rat hepatocytes. Autophagy 2, 39–46 (2006). 16874071 10.4161/auto.2229 PMC4007489

[b57] Xu C. & Webb W. Two-photon fluorescence excitation cross sections of biomolecular probes from 690 to 960 nm. J. Opt. Soc. Am. B 13, 481–491 (1996). 10.1364/ao.37.007352 18301569

[b58] Huang. H. Y. . Labile ruthenium (II) complexes with extended phenyl-substituted terpyridyl ligands: synthesis, aquation and anticancer evaluation. Dalton Trans. 44, 15602–15610 (2015). 26245234 10.1039/c5dt02446c

