# Supplementary material for: Mitochondrial Dynamics Tracking with Two-Photon Phosphorescent Terpyridyl Iridium(III) Complexes
Source: Sci Rep. 2016 Feb 11;6:20887. doi: 10.1038/srep20887 (PMC4750043; doi:10.1038/srep20887)
Supplement: Supplementary Information [file srep20887-s1.doc]

Supporting Information For

**Mitochondrial Dynamics Tracking with Two-Photon Phosphorescent Terpyridyl Iridium(III) Complexes**

**Huaiyi Huang, Pingyu Zhang, Kangqiang Qiu, Juanjuan Huang, Yu Chen,**

**Liangnian Ji and Hui Chao***

MOE Key Laboratory of Bioinorganic and Synthetic Chemistry, School of Chemistry and Chemical Engineering, Sun Yat-Sen University, Guangzhou 510275, P. R. China.

E-mail: [ceschh@mail.sysu.edu.cn](mailto:ceschh@mail.sysu.edu.cn)

Fax: 86-20-84112245, Tel: 86-20-84110613

**Table of Contents**

**Figure S1** ES-MS spectrum of **Ir1**…...……..…………...…..……………………...2

**Figure S2** ^1^H NMR spectrum of **Ir1**….……..…………...…..……………………...2

**Figure S3** ES-MS spectrum of **Ir2**…...……..…………...…..……………………...3

**Figure S4** ^1^H NMR spectrum of **Ir2**…………….…………….………….………..3

**Figure S5** ES-MS spectrum of **Ir3**…..……. …..……..……....…..………………..4

**Figure S6** ^1^H NMR spectrum of **Ir3**………….…….………….……………….…...4

**Figure S7** UV–Vis spectra of **Ir1**-**Ir3** (10 μM) in different solvent. ……………………..5

**Figure S8** Two-photon absorption cross-section of **Ir1-Ir3**……………………………….6

**Figure S9** Luminescent spectra of **Ir1**-**Ir3** (10 μM) in different solvent.…………………7

**Figure S10** Stability test of **Ir1-Ir3** in PBS buffer for 0 h and 24 h ………………………8

**Figure S11** Colocalization images of **Ir1-Ir3** with LTR…. …..…………..……....………9

**Figure S12** ICP-MS assays for the distribution of **Ir1-Ir3**…………………………………9

**Figure S13** Percentage of cell viability after incubation with **Ir1-Ir3**……………………10

**Figure S14** Images of living HeLa cells incubated with different concentrations of **Ir1-Ir3**…10

**Figure S15** Cellular uptake mechanism of **Ir1-Ir3** in present of inhibitors..……….……...11

**Figure S16** Two-photon confocal images of cells incubated with **Ir1-Ir3** and MTR………...11

**Figure S17** The axial OPM and TPM phosphorescence of **Ir1** on intact 3D MCTSs.………..12

**Figure S18** The axial OPM and TPM phosphorescence of **Ir2** on intact 3D MCTSs. ……..12

**Figure S19** The axial OPM and TPM phosphorescence of MTR on intact 3D MCTSs….…...13

**Table S1** Photophysical properties of **Ir1-Ir3** …………. ……. …….……….……14


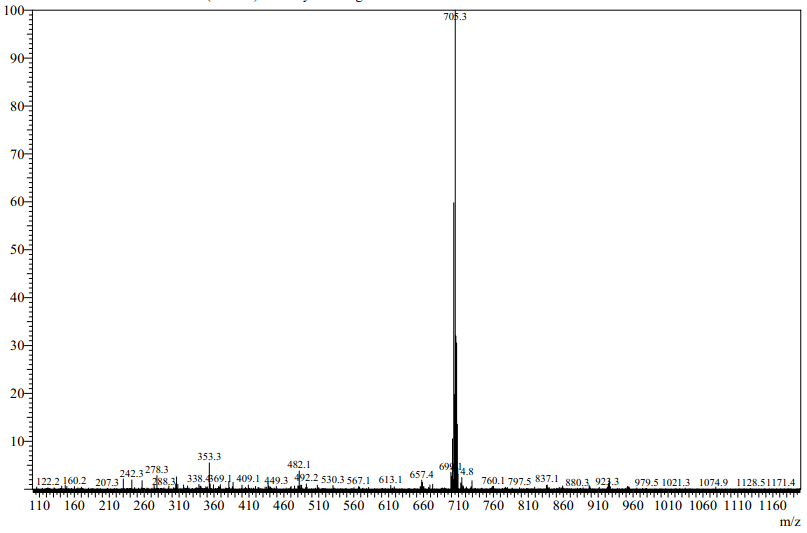


**Figure S1** ES-MS (CH_3_OH) spectrum of **Ir1**.


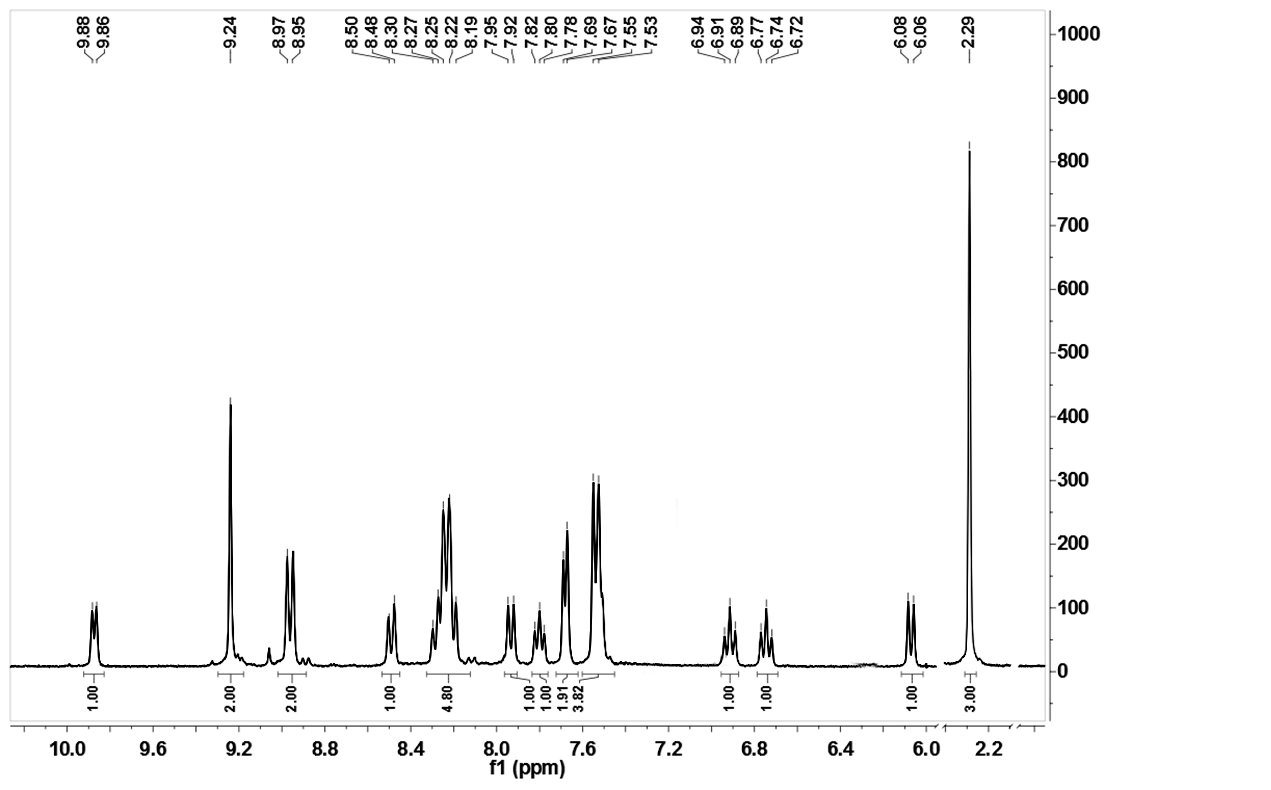


**Figure S2** ^1^H NMR (300 MHz, DMSO-*d*_6_) spectrum of **Ir1**.


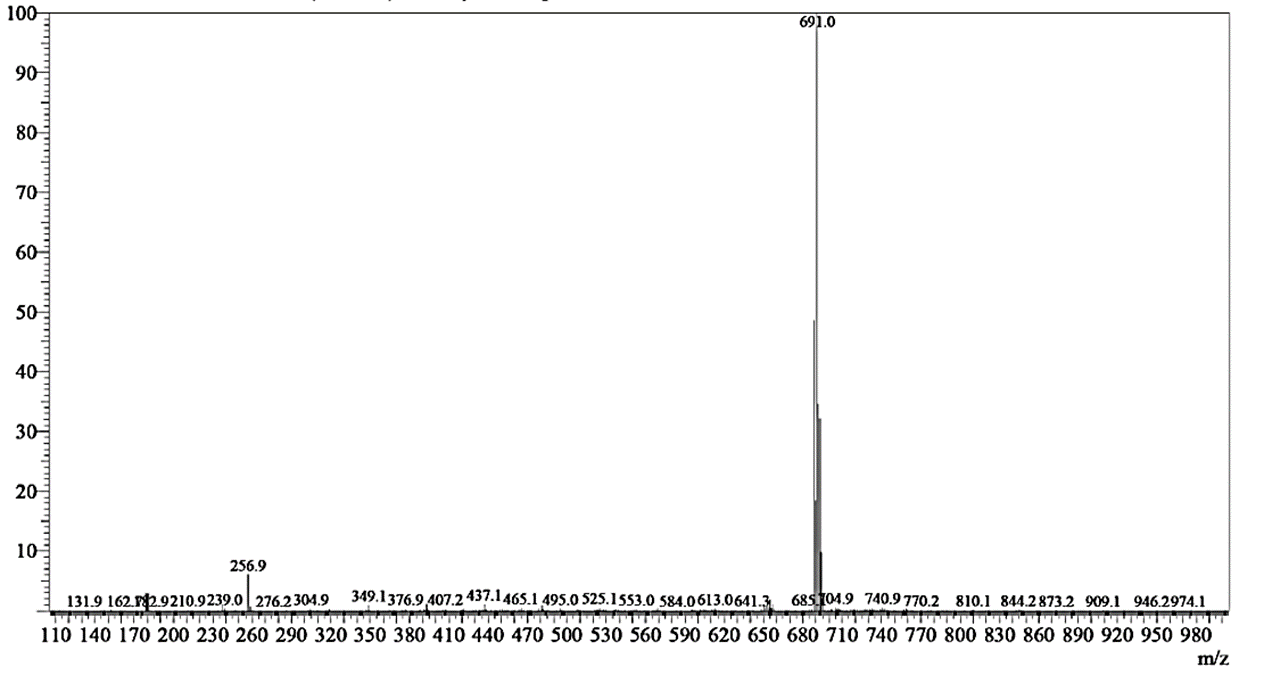


**Figure S3** ES-MS (CH_3_OH) spectrum of **Ir2**.


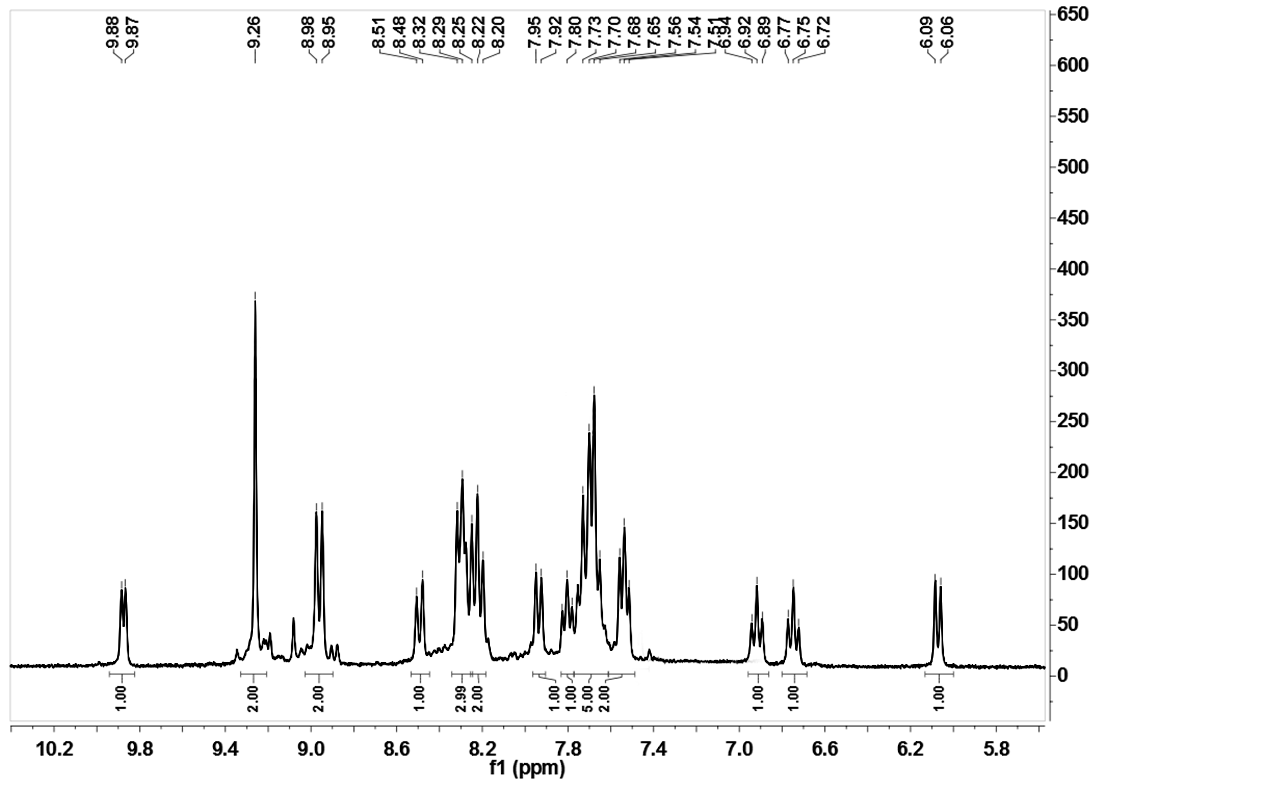


**Figure S4** ^1^H NMR (300 MHz, DMSO-*d*_6_) spectrum of **Ir2**.


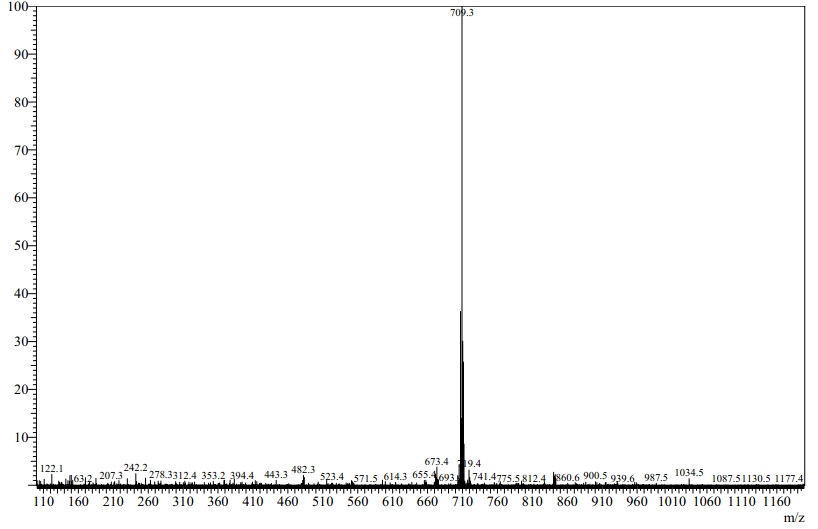


**Figure S5** ES-MS (CH_3_OH) spectrum of **Ir3**.


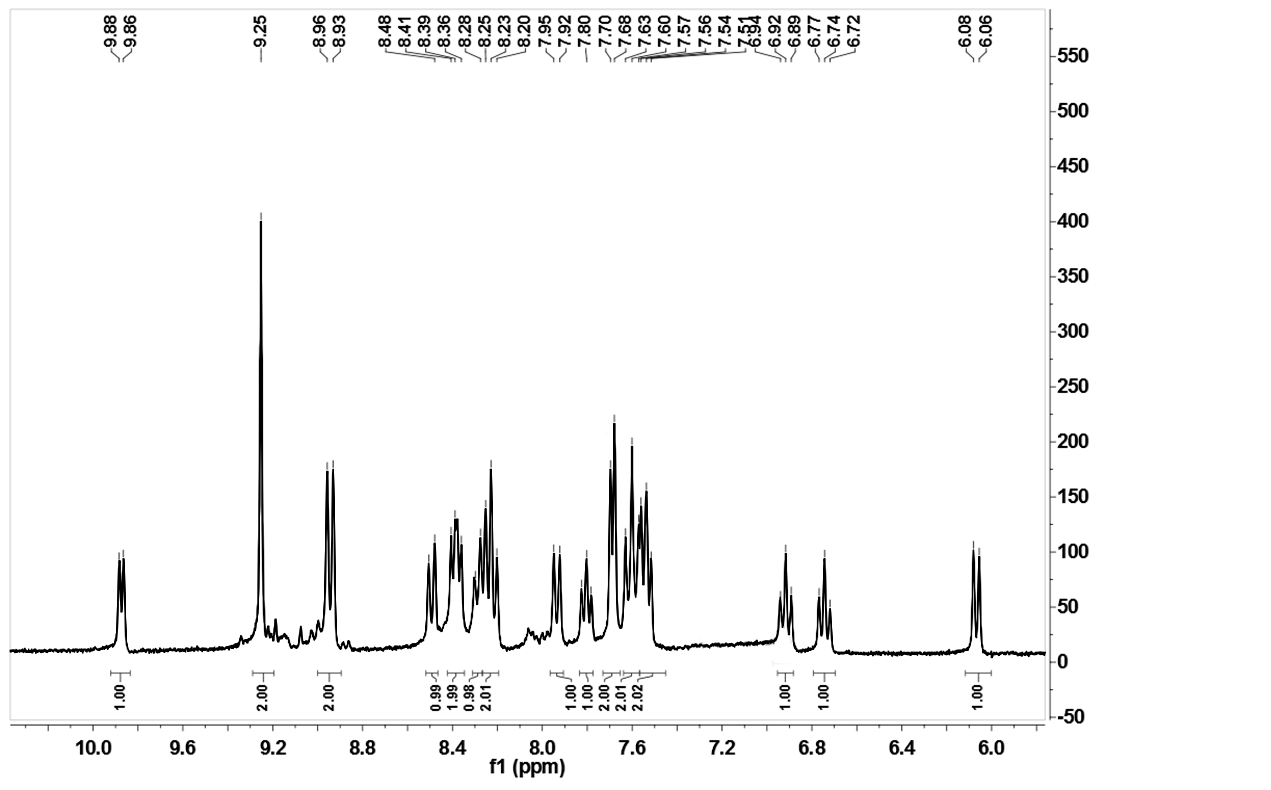


**Figure S6** ^1^H NMR (300 MHz, DMSO-*d*_6_) spectrum of **Ir3**.
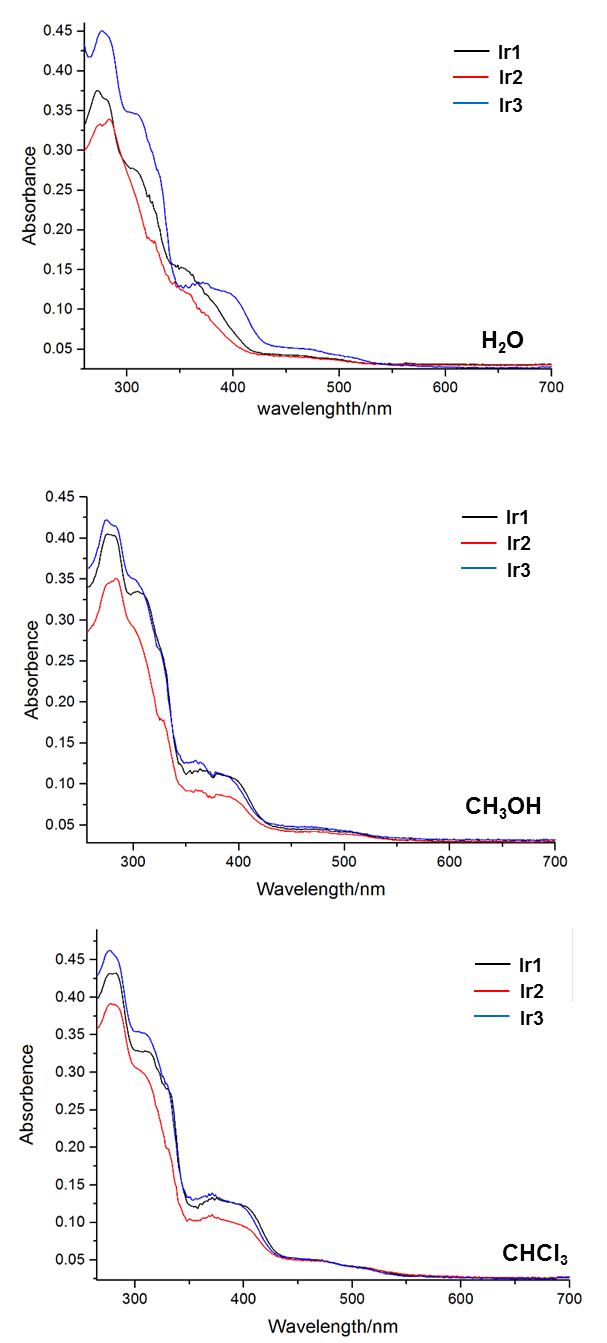


**Figure S7** UV–Vis spectra of **Ir1**-**Ir3** (10 μM) in different solvent.


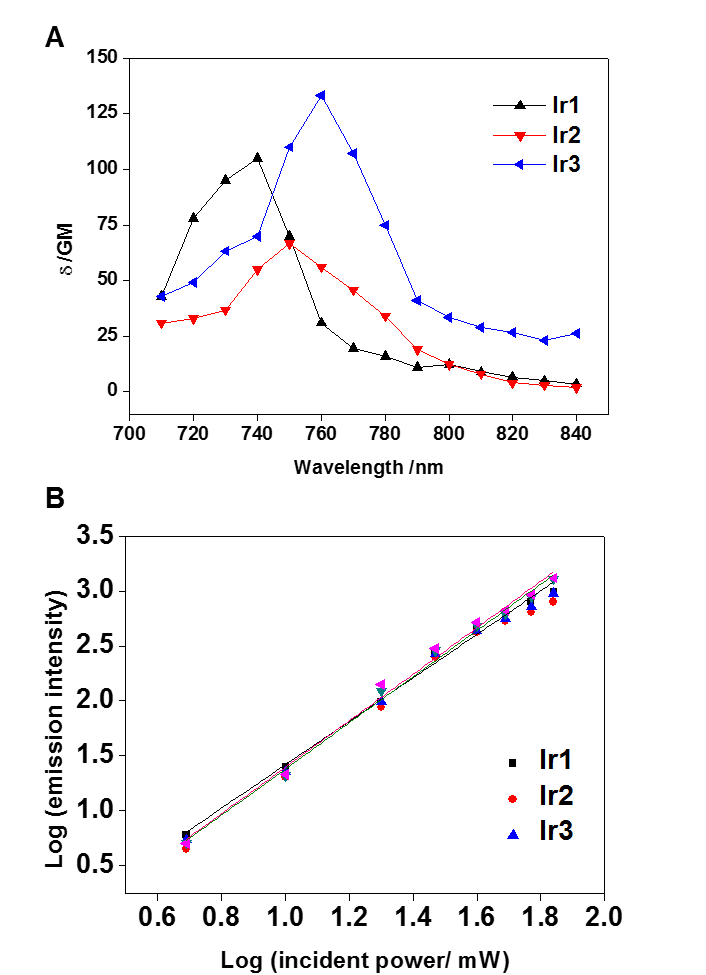


**Figure S8** (A) Two-photon absorption cross-section of **Ir1-Ir3** using excitation wavelengths from 700 to 900 nm in CH_3_OH. (B) The integrated emission intensity as a function of laser power for **Ir1-Ir3**. The plot showed quadratic dependence of the integrated emission intensity on the excitation intensity, confirming the nonlinear absorption characteristics of **Ir1-Ir3**.


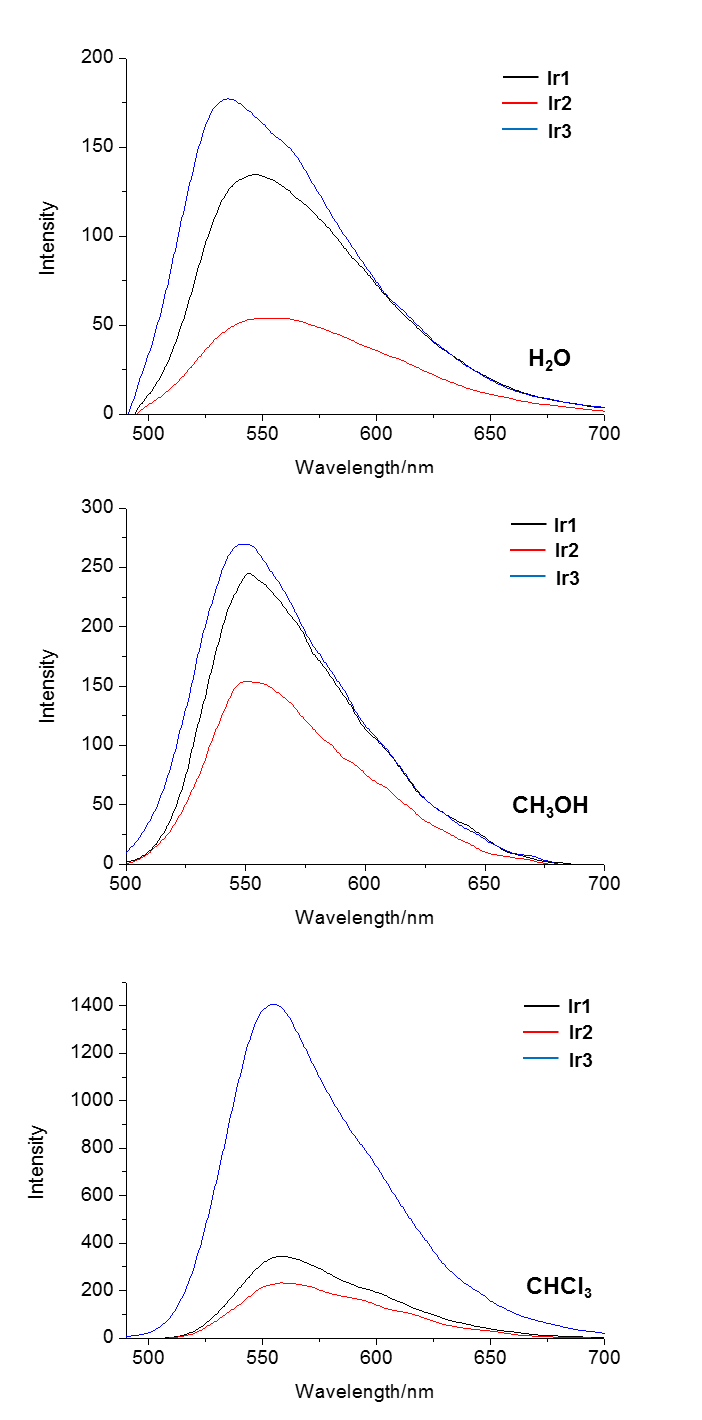


**Figure S9** Luminescent spectra of **Ir1**-**Ir3** (10 μM) in different solvent.


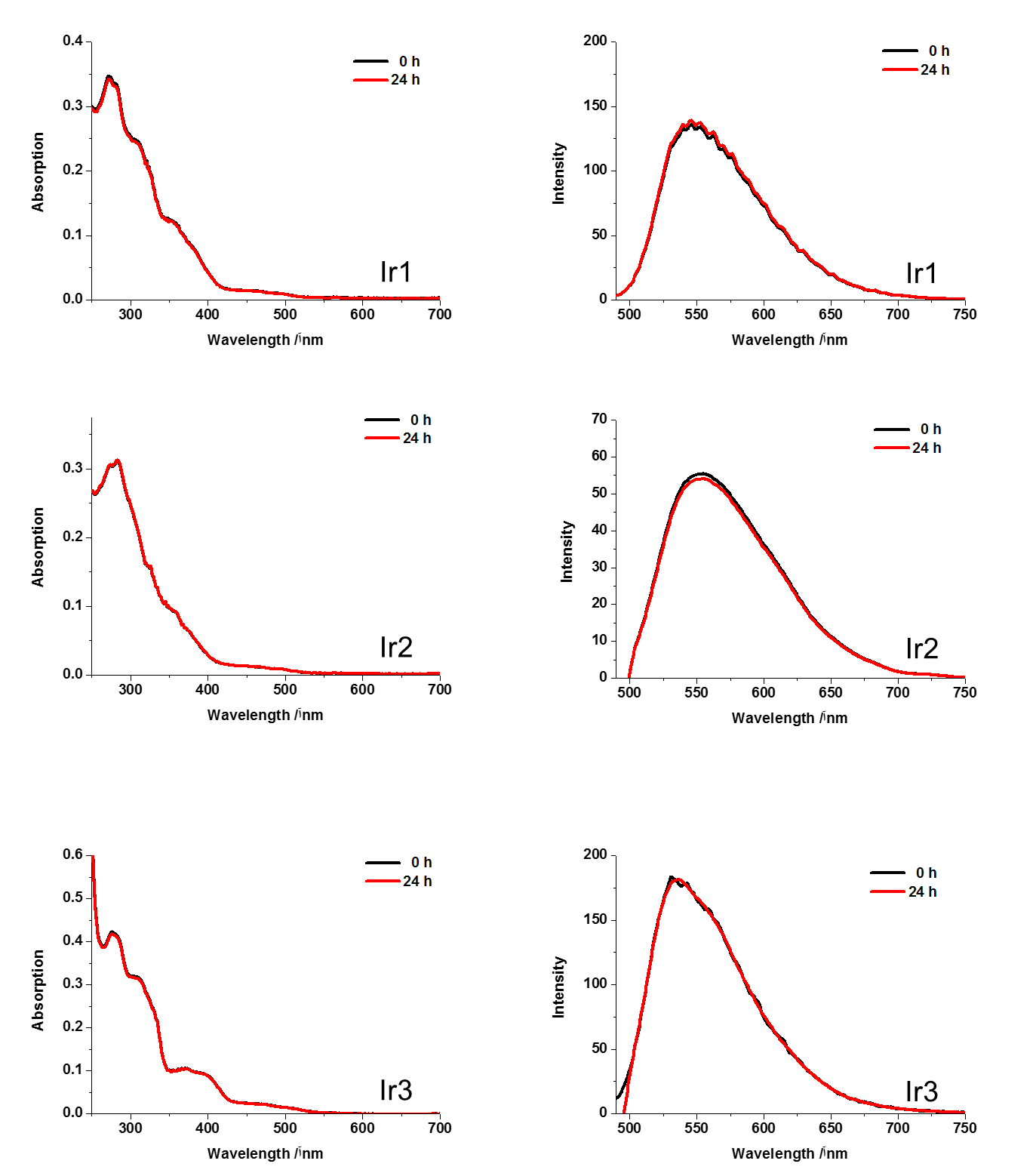


**Figure S10** Stability test of **Ir1-Ir3** in PBS buffer for 0 h and 24 h.


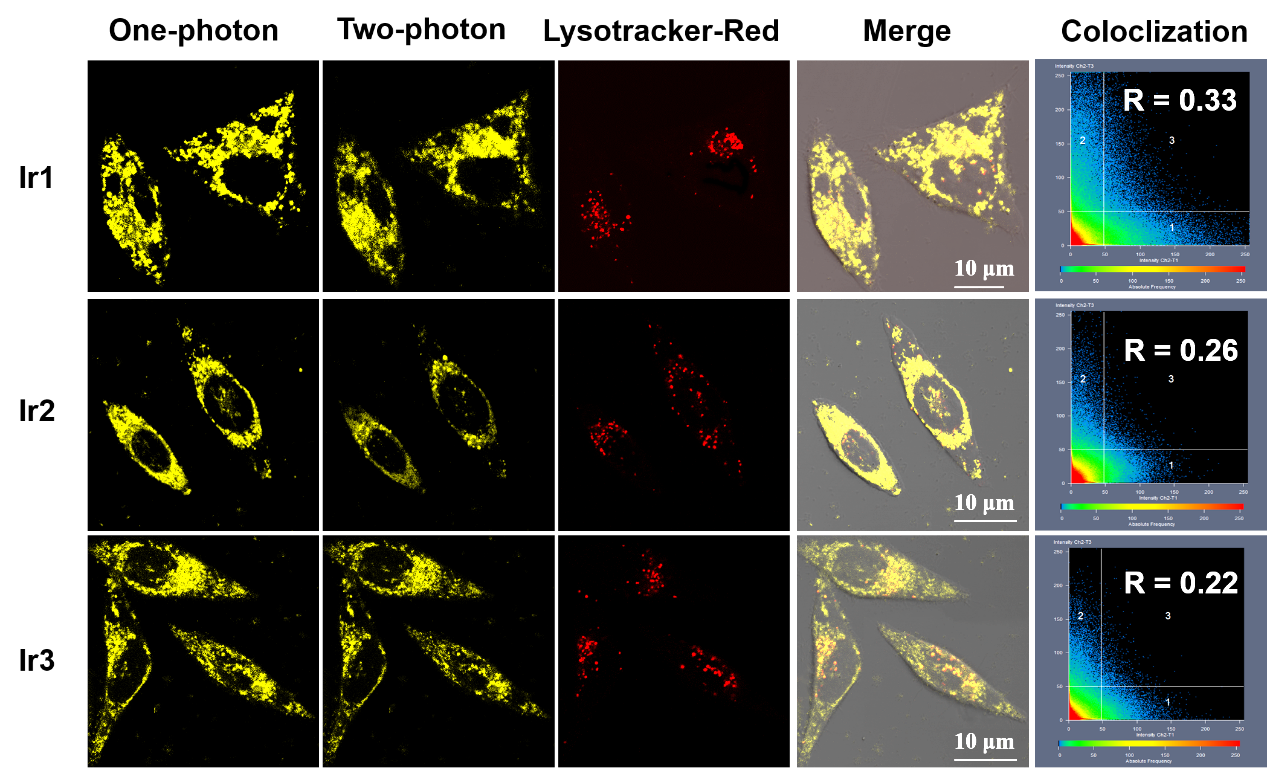


**Figure S11** Colocalization images of **Ir1-Ir3** with LTR and the colocalization coefficient (R). The excitation wavelength for **Ir1-Ir3** is 458 nm. The excitation wavelength of MTR is 543 nm. Emission ﬁlter: Emission ﬁlter: 552 ± 20 nm (for **Ir1**), 547 ± 20 nm (for **Ir2**), 535 ± 20 nm (for **Ir3**), 610 ± 20 nm (for MTR).


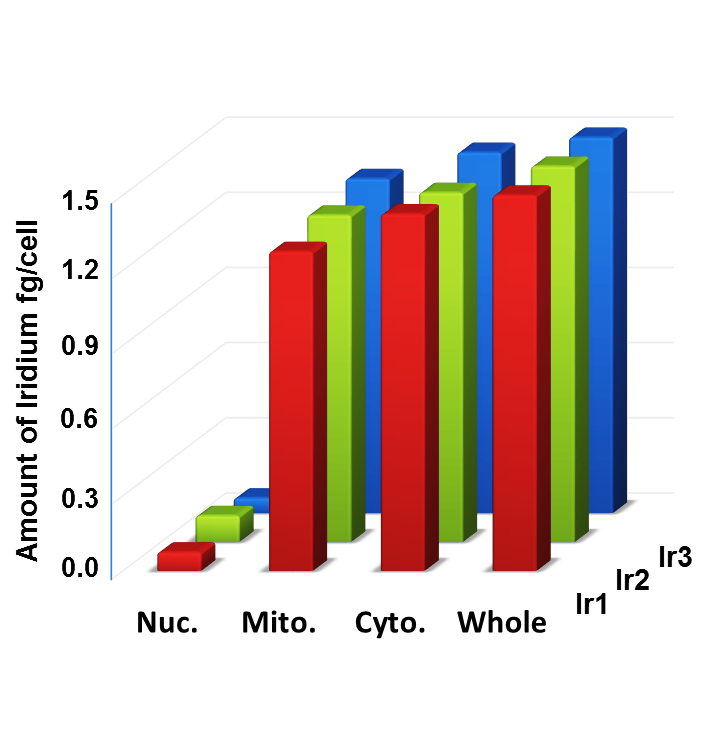


**Figure S12** ICP-MS assays for the distribution of **Ir1-Ir3** (5.0 μM) within different cellular compartments of HeLa cells.

**Figure S13** Percentage of cell viability after incubation with **Ir1-Ir3** (5 μM).


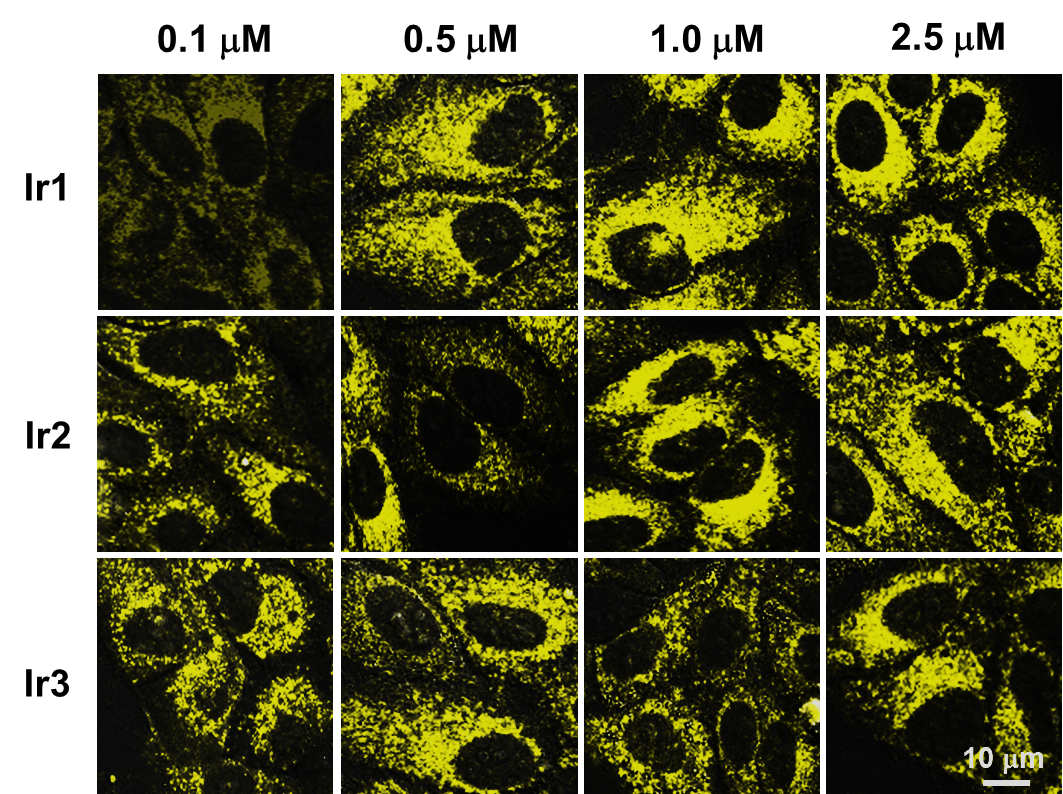


**Figure S14** Confocal images of living HeLa cells incubated with different concentrations of **Ir1-Ir3**. The excitation wavelength for **Ir1-Ir3** is 458 nm. Emission ﬁlter: Emission ﬁlter: 552 ± 20 nm (for **Ir1**), 547 ± 20 nm (for **Ir2**), 535 ± 20 nm (for **Ir3**).


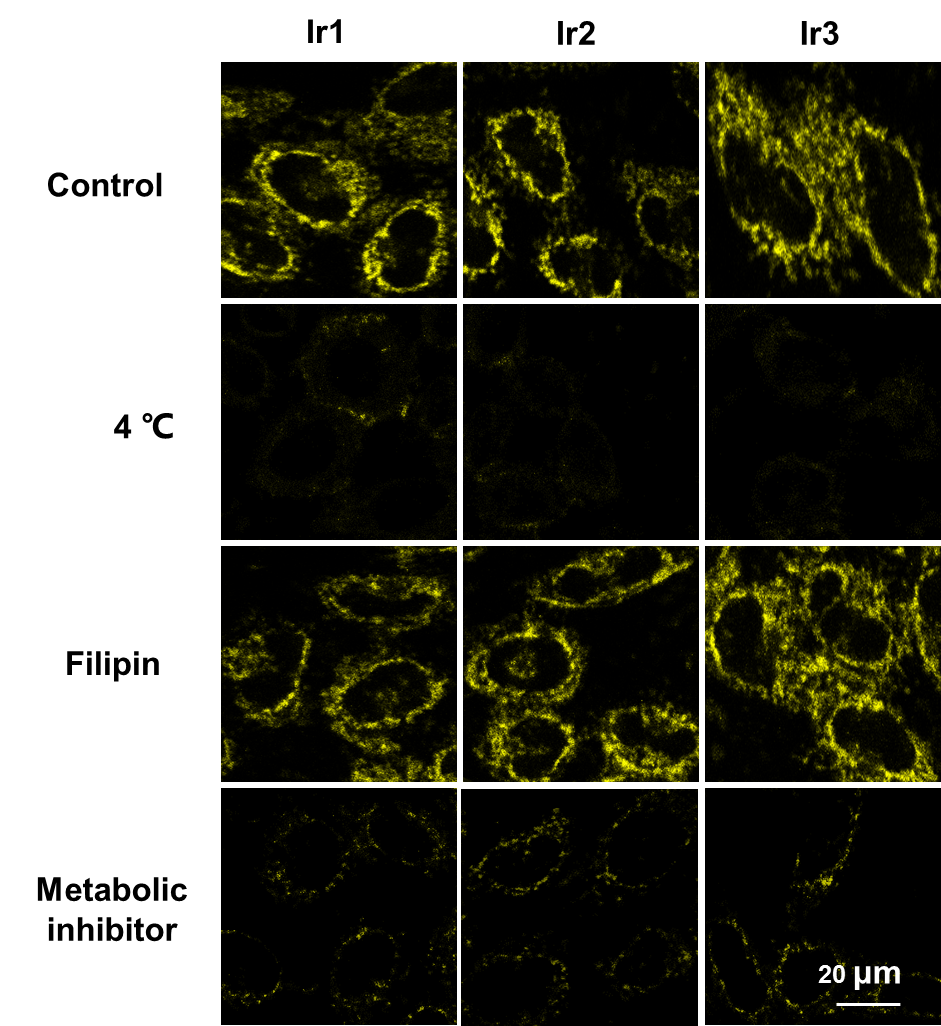


**Figure S15** Probing the cellular uptake mechanism of **Ir1-Ir3** in present of inhibitors. HeLa cells were pretreated with endocytosis inhibitors (200 μg/mL of ﬁlipin), metabolic inhibitors (50 mM of 2-deoxy-D-glucose and 5 μM of oligomycin), or free DMEM medium followed by treatment with 5 μM of **Ir1-Ir3** for 30 min at 4 °C or 37 °C.


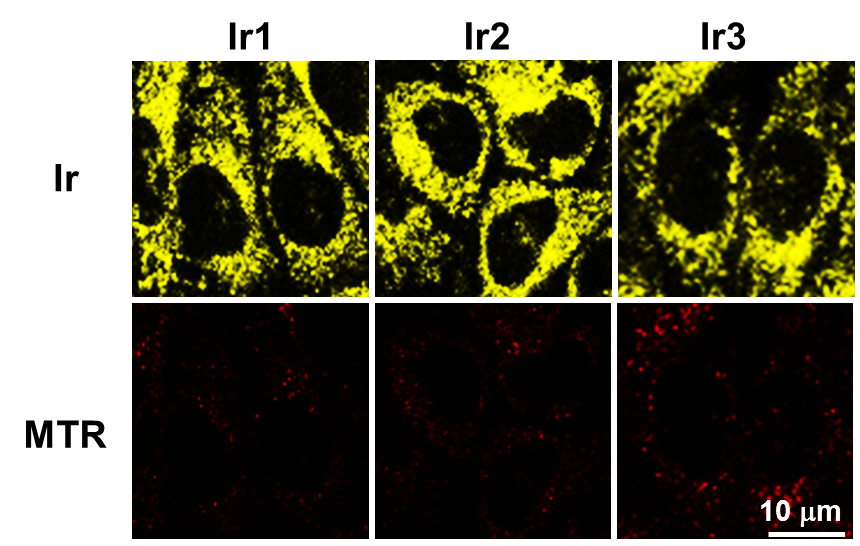


**Figure S16** Two-photon confocal images of living HeLa cells incubated with **Ir1-Ir3** and MTR. The excitation wavelength was 750 nm. Emission ﬁlter: Emission ﬁlter: 552 ± 20 nm (for **Ir1**), 547 ± 20 nm (for **Ir2**), 535 ± 20 nm (for **Ir3**), 640 ± 20 nm (for MTR).


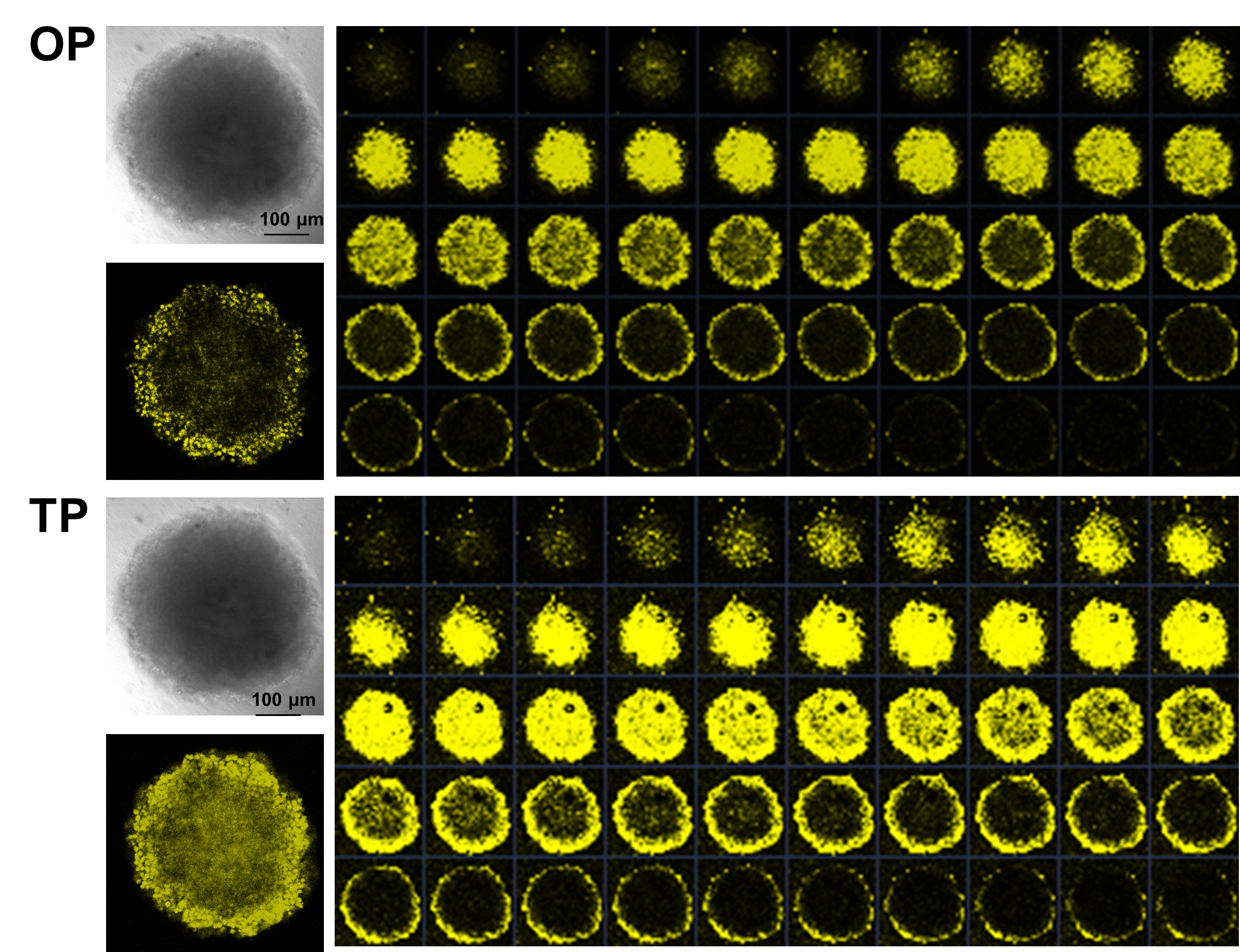


**Figure S17** The axial OPM and TPM phosphorescence of **Ir1** on intact 3D MCTSs.


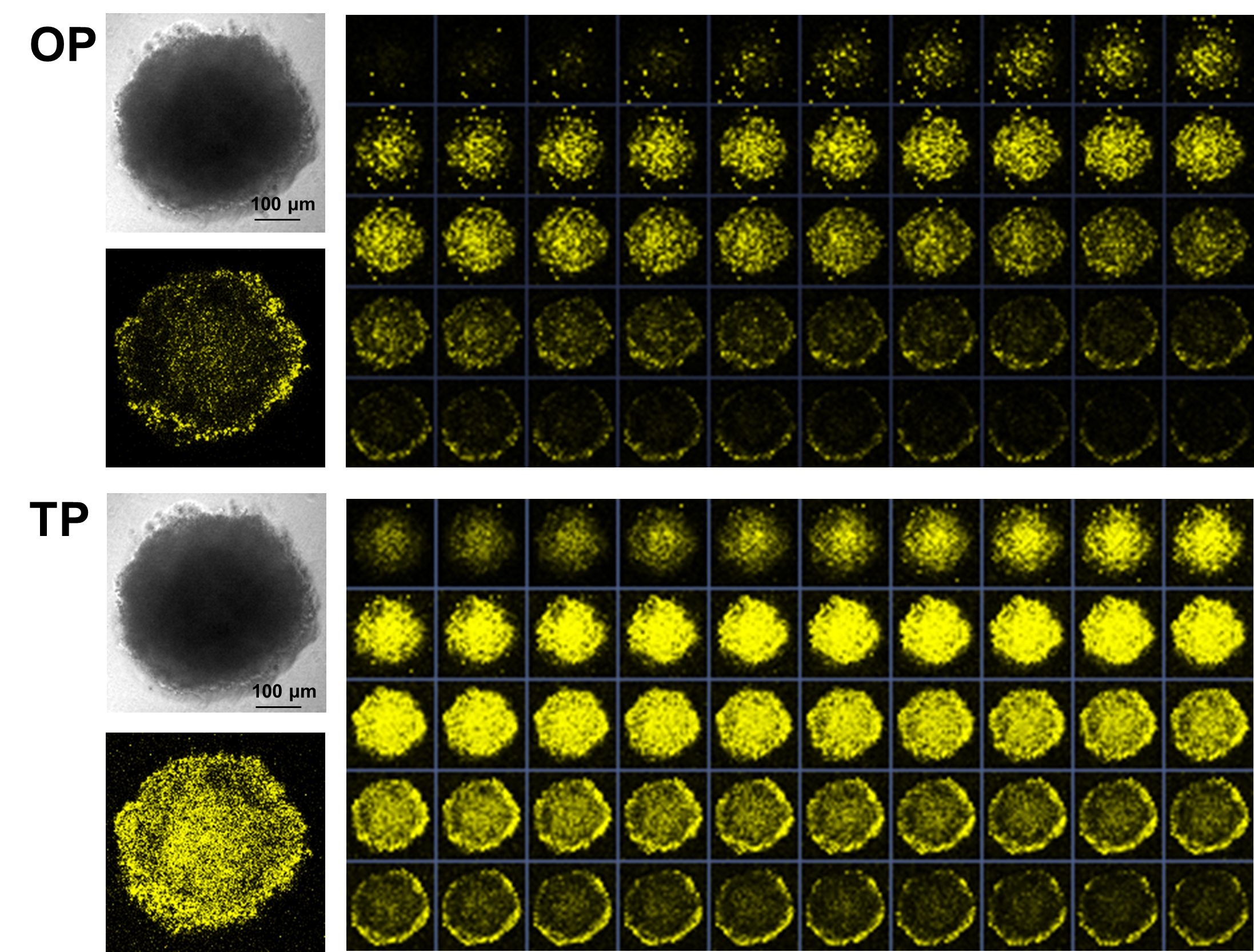


**Figure S18** The axial OPM and TPM phosphorescence of **Ir2** on intact 3D MCTSs.


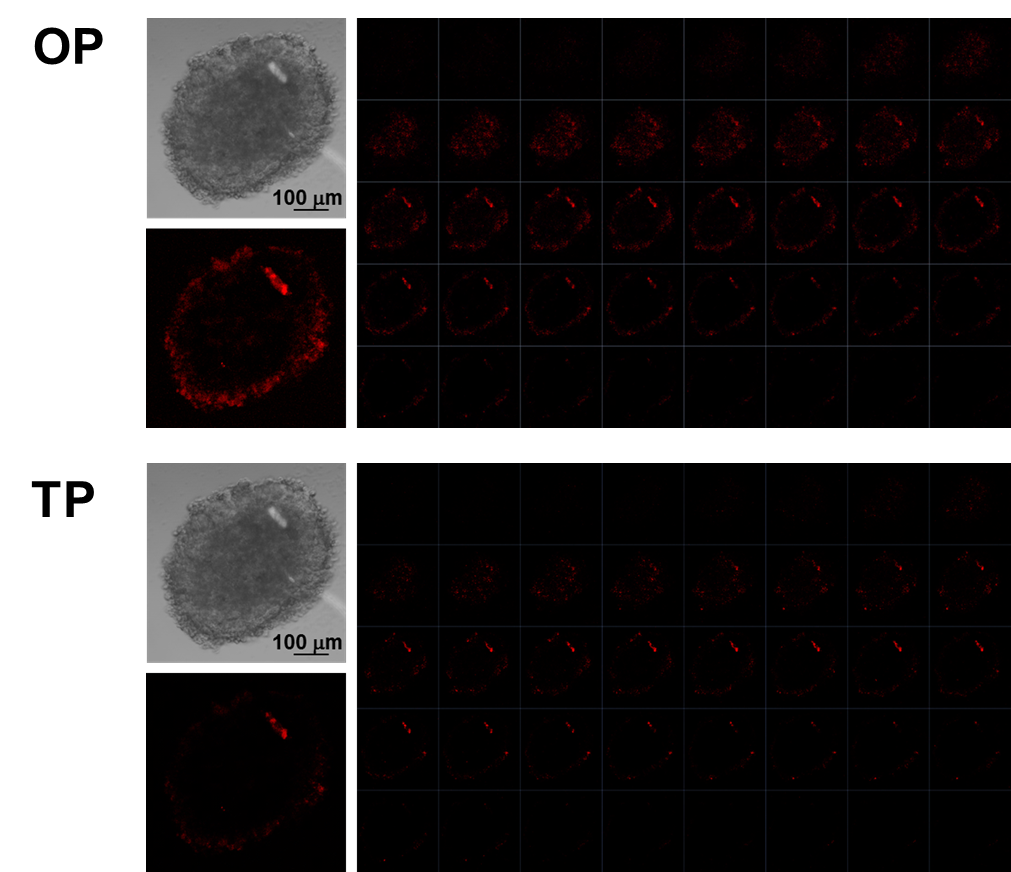


**Figure S19** The axial OPM and TPM phosphorescence of MTR on intact 3D MCTSs.

| **Table S1. Photophysical property of Ir1-Ir3.** | | | | | |
| --- | --- | --- | --- | --- | --- |
| Complex | Solvent | λ_abs_/nm (logε) | λ_PL_/nm*^a^* | Stock shift /nm*^a^* | Φ_em_ |
| **Ir1** | H_2_O | 284(3.52), 320(3.45), 350 (3.17), 458(2.87) | 552 | 94 | 0.0765 |
|  | CH_3_OH | 283(3.60), 322(3.51), 350 (3.04), 458(3.02) | 552 | 94 | 0.1082 |
|  | CHCl_3_ | 277(3.62), 320(3.51), 350 (3.07), 458(3.05) | 558 | 100 | 0.0851 |
| **Ir2** | H_2_O | 277(3.56), 320(3.35), 350 (3.09), 458(2.77) | 547 | 89 | 0.1062 |
|  | CH_3_OH | 276(3.54), 319(3,43), 350 (2.95), 458(2.91) | 550 | 92 | 0.1118 |
|  | CHCl_3_ | 276(3.59), 320(3.46), 350 (3.00), 458(3.01) | 559 | 101 | 0.1619 |
| **Ir3** | H_2_O | 279(3.65), 320(3.49), 350 (3.10), 458(3.07) | 535 | 77 | 0.1185 |
|  | CH_3_OH | 277(3.62), 319(3.54), 350 (3.11), 458(3.04) | 548 | 90 | 0.1078 |
|  | CHCl_3_ | 278(3.66), 320(3.54), 350 (3.11), 458(3.11) | 555 | 97 | 0.1973 |
